# Protein characterization of the IgM triplet involved in the diagnosis of congenital toxoplasmosis

**DOI:** 10.1051/parasite/2025065

**Published:** 2025-11-26

**Authors:** Maria Carreno, Odile Villard, Isabelle Villena, Lucie Peyclit, Helene Diemer, Christine Schaeffer-Reiss, Coralie L’Ollivier

**Affiliations:** 1 IHU Méditerranée Infection 13005 Marseille France; 2 Laboratoire de Parasitologie et Mycologie Médicale, Laboratoire Associé CNR de la Toxoplasmose, Les Hôpitaux Universitaires de Strasbourg 67091 Strasbourg France; 3 Institut de Parasitologie et Pathologie Tropicale, UR7292 Dynamique des interactions hôte pathogène, Fédération de Médecine Translationnelle, Université de Strasbourg 67091 Strasbourg France; 4 UR ESCAPE, Université Reims-Champagne-Ardenne, Service de Parasitologie-Mycologie, Centre National de Référence de la Toxoplasmose, Centre Hospitalier Universitaire (CHU) Reims 51092 Reims France; 5 Laboratoire de Spectrométrie de Masse BioOrganique, Université de Strasbourg, CNRS, IPHC UMR7178 67087 Strasbourg France; 6 Infrastructure Nationale de Protéomique ProFI, UAR2048 Strasbourg France; 7 Aix Marseille Univ, IRD, AP-HM, SSA, VITROME 13005 Marseille France

**Keywords:** *Toxoplasma gondii*, Congenital toxoplasmosis, IgM triplet, Immunoproteomics, 2-DE Immunobloting, LC-MS/MS

## Abstract

Congenital toxoplasmosis is a fetal infection resulting from the transplacental transmission of *Toxoplasma gondii* in mothers who seroconvert during pregnancy. Neonatal diagnosis has recently been improved through the identification by L’Ollivier *et al.* (2012) and Peyclit *et al.* (2023) of a pathognomonic marker for congenital toxoplasmosis: the IgM triplet, corresponding to three high molecular weight bands of 75, 90, and 100 kDa, respectively found on the mother-child immunoblot pair profile. This is a new concept, as these three IgM bands reflect an immune response targeting proteins involved in vertical transmission of *T. gondii*. These proteins may be *T. gondii* secreted or non-secreted effectors implicated in host cell invasion, immune modulation, and parasite virulence. In this study, immunoproteomic techniques allowed us to identify thirty-two relevant protein spots on immunoblot, including four specifically associated with the IgM triplet. Protein identification by LC-MS/MS revealed several *T. gondii* proteins as strong candidates for the IgM triplet. Each of these proteins is, directly or indirectly, involved in cellular invasion and may also play a role in transplacental transmission of *T. gondii*. Identifying these proteins opens several avenues of therapeutic research that could improve the management of congenital toxoplasmosis.

## Abstract (fr)

La toxoplasmose congénitale est une infection fœtale résultant de la transmission transplacentaire de *Toxoplasma gondii* chez des mères séroconverties pendant la grossesse. Le diagnostic néonatal a récemment été amélioré grâce à l’identification par L’Ollivier *et al.* (2012) et Peyclit *et al.* (2023) d’un marqueur pathognomonique de la toxoplasmose congénitale : la triplette IgM, correspondant à trois bandes de haut poids moléculaire de 75, 90 et 100 kDa respectivement, présentes sur le profil de paires d’immunoblots mère-enfant. Il s’agit d’un concept nouveau, car ces trois bandes IgM reflètent une réponse immunitaire ciblant des protéines impliquées dans la transmission verticale de *T. gondii*. Ces protéines pourraient être des effecteurs sécrétés ou non par *T. gondii* impliqués dans l’invasion des cellules hôtes, la modulation immunitaire et la virulence du parasite. Dans cette étude, les techniques d’immunoprotéomique nous ont permis d'identifier trente-deux spots protéiques pertinents sur immunoblot, dont quatre spécifiquement associés à la triplette IgM. Pour ces spots, l'identification des protéines par LC-MS/MS a révélé que plusieurs protéines de *T. gondii* étaient de bonnes candidates pour la triplette IgM. Chacune de ces protéines est impliquée, directement ou indirectement, dans l’invasion cellulaire et pourrait également jouer un rôle dans la transmission transplacentaire de *T. gondii*. L’identification de ces protéines ouvre de nouvelles perspectives thérapeutiques susceptibles d’améliorer la prise en charge de la toxoplasmose congénitale.

## Introduction

*Toxoplasma gondii* (*T. gondii)* is a ubiquitous pathogen that can infect a wide range of hosts, such as the majority of birds and mammals (including humans). In humans, infection is usually asymptomatic or causes a simple flu-like illness in immunocompetent patients, but can cause severe disease in immunocompromised patients and complications in newborns (NB) if the mother seroconverts during pregnancy (congenital toxoplasmosis [CT]) [13]. Indeed, CT can lead to serious complications in the fetus and NB, including abnormal psychomotor development, ocular lesions such as chorioretinitis, and neurological disorders [19].

Diagnosis of CT is based on prenatal screening and/or the detection of neosynthesized IgG, IgM, or IgA in the NB, and/or IgG persisting for 12 months after birth. Treatment of the infected infant should be initiated as early as possible to minimize clinical sequelae. It is recognized that the IgG/IgM mother-infant immunoblot pair profiles (IbPP) help in early neonatal diagnosis. L’Ollivier *et al.* [14] and Peyclit *et al.* have also demonstrated that the presence of three IgM bands in the infant profile, at 75, 90, and 100 kDa, is pathognomonic for the diagnosis of CT (Supplementary data S1) [14, 21]. Proteomic characterization of this IgM triplet would enhance our understanding of its significance in CT diagnosis and provide insights into the pathophysiological mechanisms underlying transplacental transmission [2].

The precise mechanisms of *T. gondii* vertical transmission remain poorly understood due to the lack of laboratory models reflecting the complexity of the maternal-fetal interface [1]. Nevertheless, based on literature data, assumptions can be made regarding the parasite’s ability to reach the intra-amniotic compartment. It has to overcome both mechanical barriers, with the syncytiotrophoblasts as the main layer to be crossed, and immunological barriers from both the maternal and fetal immune systems. As a result, *T. gondii* has developed a complex set of molecular mechanisms involving secreted and non-secreted proteins to invade and manipulate host cells, thereby contributing to its virulence. These proteins play a role throughout the parasite’s lytic cycle and may be involved in maternal-fetal transmission of *T. gondii* across the placental barrier [2].

In this study, we used immunoproteomics techniques (two-dimensional electrophoresis [2-DE], immunoblotting, and liquid chromatography coupled to tandem mass spectrometry [LC-MS/MS]) to identify the proteins targeted by the IgM triplet using sera from NBs with confirmed CT who exhibited this triplet in their mother-infant IbPP.

## Materials and methods

### Ethics approval

The study was conducted in accordance with the tenets of the Declaration of Helsinki and approved by the Ethics Committee of the Assistance Publique des Hôpitaux de Marseille, France (protocol code AC-2009-1031 of July 27, 2010, and protocol code 2019-73 of May 29, 2019).

### IgM triplet control sera

The selected serum or cord blood samples were obtained from the routine diagnostic activity of a parasitology-mycology laboratory (IHU Méditerranée, Marseille, France). Sera were stored at −20 °C and selected between 2010 and 2021. Sixteen serum samples from NBs with proved CT were selected based on the presence of the IgM triplet on the IbPP performed at birth (NNTPos group). Three serum or cord blood samples from NBs with proven CT and positive IgM, but without the IgM triplet on the IbPP at birth were selected as negative controls (NNTNeg group).

### Preparation of *T. gondii* lysate antigen

Tachyzoites of the RH strain of *T. gondii* were maintained in Swiss Webster mice (CERJ, Cergy-Pontoise, France) by intraperitoneal inoculation. Three days later, the mice were sacrificed by inhalation of isoflurane (Forène^®^, Abbott France, Rungis, France), and the tachyzoites were recovered by instilling 5 mL of sterile 0.9% NaCl solution into the peritoneal cavity. Parasites were then washed twice in phosphate-buffered saline (PBS) and used to prepare the *T*. *gondii* lysate antigen (TLA) with deoxycholate 2.5% (DOC) and sodium dodecyl sulfate 2.5% (SDS).

TLA was supplemented with a protease inhibitor cocktail (Sigma, St. Louis, MO, USA) and stored at −80 °C until purification. TLA was purified using a 2-D Clean Up Kit (GE Healthcare Bioscience, Chalfont St. Giles, United Kingdom), according to the supplier’s protocol. Proteins were quantified using the Bradford assay (Bio-Rad, Inc., Hercules, CA, USA).

### Two-dimensional electrophoresis of TLA

An amount of 20 μg of purified TLA was loaded onto each Immobiline™ Drystrip 7 cm pH 3–10 gels (IPG strips) (GE Healthcare, Uppsala, Sweden) after strip rehydration into TS buffer (7M urea, 2M thiourea, 4% CHAPS) containing 60 mM dithiothreitol (DTT) and 0.5% immobilized pH gradient buffer (pH 3-10, GE Healthcare, Uppsala, Sweden). Isoelectric focusing (IEF) was performed according to a multi-step protocol: 300 V for 30 min in linear voltage mode, 1000 V for 30 min, 5000 V for 80 min, then 5000 V for 40 min in rapid voltage mode at 20 °C using an Ettan IPG Phor II IEF system (GE Healthcare). Upon completion of the IEF, the IPG strips were removed from the device and stored at −20 °C before the next step. IPG strips were then equilibrated for 15 min in equilibration buffer (1.5 M Tris-HCl, pH 8.8, 6 M urea, 30% glycerol, 2% SDS, and 0.04% bromophenol blue) containing 65 mM DTT, then for 15 min in the same buffer used for DTT plus 100 mM iodoacetamide. For two-dimensional electrophoresis (2-DE), proteins were separated by SDS-PAGE electrophoresis on 12% resolving gels. Electrophoresis was performed at 30 mA for 70 min. Next, 2-DE gels were fixed in 40% ethanol and 10% glacial acetic acid for 12 h and then stained with silver nitrate to visualize all the proteins of *T. gondii*. Silver-stained gels were scanned using ImageScanner III (EPSON Scan) and Image Ready software (v 7.0.1, Adobe Systems).

Thirty-eight 2-DE gels were reserved for IgM triplet revelation using the selected NB sera. The 16NNTPos sera and the 3 NNTNeg sera were for immunoblot analysis in duplicate.

### Immunoblot analysis using selected sera

Protein on the 2-DE gels were transferred to nitrocellulose membranes (Fisher Scientific, Illkirch, France) previously activated with 20% ethanol for optimal protein transfer. Transfer was performed at 100 V for 90 min at 4 °C. After transfer, the membranes were blocked with a saturation buffer containing 5% skim milk in PBS with 0.3% Tween 20 (PBST) for 1 h at room temperature. The membranes were then incubated overnight at 4 °C with NNTPos or NNTNeg sera diluted at 1:50.

After three 10-minute washes in PBST, the transfers were incubated with peroxidase-conjugated goat anti-human IgM secondary antibodies (Jackson ImmunoResearch, Philadelphia, PA, USA) diluted 1:5000 in PBST-5% skim milk for 1 h at room temperature. After three 10-minute washes in PBST, protein spots were revealed using the Clarity Western ECL Substrate Kit (Bio-Rad, Hercules, CA, USA) via the Fusion FX digital imaging system (Vilber Lourmat, Collégien, France). The images obtained were analyzed using Photoshop software (v 7.0.1, Adobe Systems).

### Determination of IgM triplet-specific proteins by liquid chromatography-mass spectrometry

The two protein profiles of silver nitrate-stained tachyzoites from the *T. gondii* RH strain were superimposed on the 32 transfers obtained from the 16 NNTPos sera analyzed in duplicate to identify any spots specific to the IgM triplet. The two protein profiles were also superimposed on the six transfers obtained from the three NNTNeg sera analyzed in duplicate to eliminate spots not specific to the IgM triplet. The spots consistently containing the immunoreactive proteins detected in the IgM triplet zone (between 75 and 100 kDa) only in NNTPos sera were excised from the two protein profiles of *T. gondii* RH strain tachyzoites stained with silver nitrate. The gel spots were destained by two washes with 50 μL of 15 mM potassium ferricyanide and 50 mM sodium thiosulfate. After three washes with water, acetonitrile, and 25 mM ammonium hydrogen carbonate (NH_4_HCO_3_), the gel plugs were dehydrated with acetonitrile. Cysteine residues were reduced with 50 μL of 10 mM dithiothreitol at 57 °C and alkylated with 50 μL of 55 mM iodoacetamide. After two washes with NH_4_HCO_3_ and acetonitrile, the gel plugs were dehydrated with acetonitrile. The digestion of proteins was done in gel with 10 μL of 7 ng/μL modified porcine trypsin (Promega, Madison, WI, USA) in 25 mM NH_4_HCO_3_. Digestion was performed overnight at room temperature. The generated peptides were extracted with 40 μL of 60% acetonitrile in 0.1% formic acid. Acetonitrile was evaporated under vacuum prior to MS analysis.

### Mass spectrometry analysis

NanoLC-MS/MS analysis was performed using a nanoACQUITY Ultra-Performance-LC (Waters Corporation, Milford, MA, USA) coupled to a Q-Exactive Plus mass spectrometer (Thermo Fisher Scientific, Bremen, Germany). The entire system was fully controlled by Thermo Scientific™ Xcalibur™ software (v3.1). Peptides were trapped on a NanoEase^TM^ M/Z Symmetry C18 precolumn (100 Å, 5 μm, 180 μm × 20 mm, Waters Corporation) using 99% solvent A (0.1% formic acid in water) and 1% solvent B (0.1% formic acid in acetonitrile) at a flow rate of 5 μL/min for 3 min. A solvent gradient from 1 to 6% of B in 0.5 min and then from 6–40% of B in 44 min was used for peptide elution, which was performed at a flow rate of 350 nL/min using a NanoEase^TM^ M/Z BEH C18 column (130 Å, 1.7 μm, 75 μm × 250 mm, Waters Corporation) maintained at 60 °C. The Q-Exactive Plus was operated in positive ion mode with a source temperature of 250 °C and a spray voltage of 1.8 kV. Full scan MS spectra (300–1800 m/z) were acquired at a resolution of 70,000 at m/z 200.

MS parameters were set as follows: maximum injection time of 50 ms, automatic gain control (AGC) target value of 3e6 ions, lock mass option enabled (polysiloxane, 445.12002 m/z), selection of up to 10 most intense precursor ions (doubly charged or more) per full scan for subsequent isolation using a 2 m/z window, fragmentation using higher-energy collisional dissociation (HCD, normalized collision energy of 27), dynamic exclusion of already fragmented precursors set to 60 s. MS/MS spectra (200–2000 m/z) were acquired with a resolution of 17,500 at m/z 200. MS/MS parameters were set as follows: maximum injection time of 100 ms, AGC target value of 5e3 ions, peptide match selection option turned on. Raw data were converted into mgf files using the MSConvert tool from ProteomeWizard (v3.0.6090; http://proteowizard.sourceforge.net/).

### Protein identification

For protein identification, MS/MS data were searched using a local Mascot server (v2.6.2; Matrix Science, London, United Kingdom) against a database containing the sequences of all *T. gondii ME49* entries as found in https://toxodb.org/ (ToxoDB-10.0_TgondiiME49_AnnotatedProteins.fasta, 8,322 sequences) and the corresponding 8,322 reverse sequences. Spectra were searched with a mass tolerance of 10 ppm for MS and 0.05 Da for MS/MS data, allowing a maximum of one missed trypsin cleavage. Carbamidomethylation of cysteine residues and oxidation of methionine residues were specified as variable modifications. Identification results were imported into Proline v2.1 (www.profiproteomics.fr/proline) for validation with the following criteria: peptide spectrum matches (PSM) with pretty rank equal to 1 and a minimum sequence length of 7 were retained and the false discovery rate was then optimized to be less than 1% at the PSM level using the Mascot adjusted E-value and less than 1% at the protein level using the Mascot score.

The mass spectrometry proteomics data have been deposited to the ProteomeXchange Consortium via the PRIDE [20] partner repository with the dataset identifier PXD060424 and DOI: https://doi.org/10.6019/PXD060424.

## Results

### Delineation of the IgM triplet zone

**Figure 1:**
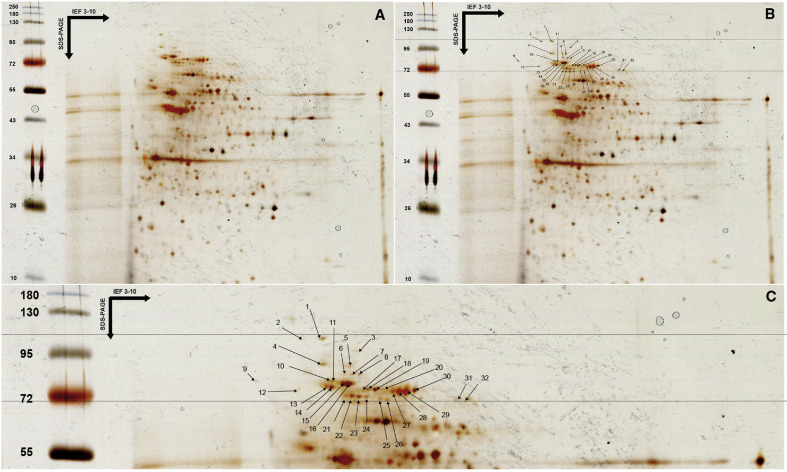
2-DE gel obtained with *T. gondii* lysate antigen after silver nitrate staining. A. All proteins from *T. gondii* RH strain tachyzoites were separated by isoelectric focusing (pH 3–10), followed by SDS-PAGE electrophoresis on polyacrylamide gel (12% acrylamide), and then revealed by silver nitrate staining. B. Delineation of the IgM triplet zone between 75 and 100 kDa and numbering of all protein spots present. C. Close-up of the protein spots in the IgM triplet zone.

Using the two protein profiles of the *T. gondii* RH strain obtained from 2-DE gels and stained with silver nitrate, 32 spots were identified in the IgM triplet zone between 75 and 100 kDa (Fig. 1). These spots appeared as brown and of varying intensities. Some spots were isolated (spots 1, 2, 3, 4, 5, 9, and 12), while others were clustered (spots 6–8, spots 10–11, spots 13–14, spots 15–16, spots 17–20, spots 21–24, spots 25–26, spots 27–30, and spots 31–32). Clustered spots may represent different proteins or the same protein with post-translational modifications.

### IgM triplet revelation using NNTPos and NNTNeg sera

**Figure 2:**
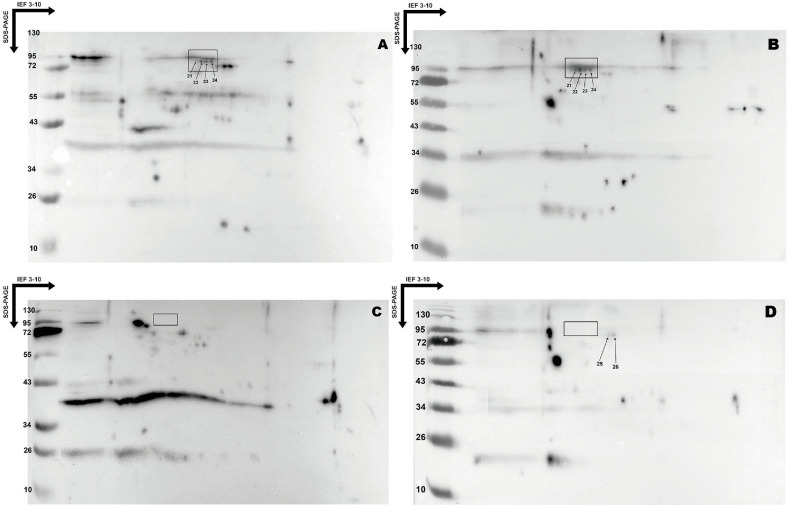
2D immunoblot of *T. gondii* RH strain proteins. A and B. Obtained after incubation with two NNTPos sera (patients 5BB (A) and 8BB (B)) and revelation with peroxidase-conjugated goat anti-human IgM antibodies. C and D. Obtained after incubation with two NNTNeg sera (patients 17BB (C) and 19BB (D)) and revelation with peroxidase-conjugated goat anti-human IgM antibodies. These immunoblots reveal the IgM triplet spots of interest (21–24) present only in NNTPos sera and identified by superposition with the protein profile of the *T. gondii* RH strain (black box). These immunoblots also make it possible to exclude the spots that are not specific to the IgM triplet (25 and 26) present in NNTPos and NNTNeg sera and identified by superposition with the protein profile of the *T. gondii* RH strain (NNTPos: newborns positive for the IgM triplet; NNTNeg: newborns negative for the IgM triplet).

A total of 32 transfers were performed for IgM triplet revelation using NNTPos sera (*n* = 16) each analyzed in duplicate. An additional six transfers were performed to exclude the spots not specific to the IgM triplet using NNTNeg sera (*n* = 3) also analyzed in duplicate (Fig. 2).

After superimposing the silver-stained protein profiles of *T. gondii* RH strain and transfers obtained with NNTPos sera using Photoshop software (v 7.0.1, Adobe), six spots previously numbered 21–26 were found to match, corresponding to one or more potential target proteins of the IgM triplet.

After superimposing the same protein profiles and patient transfers obtained with NNTNEg sera using Photoshop software (v 7.0.1, Adobe), spots 25 and 26 were excluded. Ultimately, four protein spots (21–24) were found only with the NNTPos sera (Fig. 2). The immunoblots from the 19 patients (16 NNTPos and 3 NNTNeg) are shown in the Supplementary data S2.

### Identification of IgM triplet proteins by LC-MS/MS

Protein spots to be analyzed were excised from the two silver nitrate-stained protein profiles of the *T. gondii* RH strain. A total of eight spots were collected, four from each gel as mirror images, in order to identify as many proteins as possible present in the IgM triplet zone. In the selected spots (21–24) a total of fifty-three unique proteins each with at least one matching peptide was identified by LC-MS/MS analysis. Forty proteins were identified for spot 21, twenty-nine for spot 22, thirty-three for spot 23, and thirty for spot 24 (Tables 1–4).

**Table 1:** Proteins of *Toxoplasma gondii* RH strain identified by LC-MS/MS for the first IgM triplet spot of interest (spot 21) (ND: not detected).

Protein name	Known or predicted function	Location	Accession	Organism	Molecular weight (Da)	Peptide count on protein profile No. 1	Peptide count on protein profile No. 2
Heat Shock Protein (HSP)	Molecular chaperone involved in protein folding, cellular stress responses, and regulation of host immune system	TGME49_chrXII:5582774-5587740(−)	TGME49_251780	Toxoplasma_gondii_ME49	78251	56	35
Heat Shock Protein 70 (HSP70)	Molecular chaperone involved in protein folding, cellular stress responses, and regulation of host immune system (modulation of nitric oxide production and reduction of macrophages phagocytosis capacity)	TGME49_chrVIII:3289396-3291420(−)	TGME49_273760	Toxoplasma_gondii_ME49	72880	53	51
Chaperonin protein BiP	Molecular chaperone involved in protein folding and exit from the endoplasmic reticulum	TGME49_chrXI:2225378-2228753(−)	TGME49_311720	Toxoplasma_gondii_ME49	73253	43	26
Heat Shock Protein 90 (HSP90)	Molecular chaperone involved in protein folding, cellular stress responses, and regulation of host immune system	TGME49_chrIX:2483404-2487798(+)	TGME49_288380	Toxoplasma_gondii_ME49	81933	32	30
Alveolin domain containing intermediate filament IMC1 (ALV1)	Inner membrane complex protein associated with the membrane cytoskeleton of Toxoplasma gondii, involved in maintaining parasite morphology and motility	TGME49_chrVIII:1516857-1519632(+)	TGME49_231640	Toxoplasma_gondii_ME49	69596	25	14
Peptidase M16 inactive domain-containing protein	Member of the M16A metalloprotease family, no function described	TGME49_chrX:716364-726488(+)	TGME49_227948	Toxoplasma_gondii_ME49	142764	13	2
Tryptophanyl-tRNA synthetase (TrpRS2)	Apicoplast protein involved in the aminoacylation of tRNA during proteins synthesis	TGME49_chrIX:2473710-2478137(+)	TGME49_288360	Toxoplasma_gondii_ME49	77058	10	9
Toxolysin 4 (TLN4)	Member of the M16A metalloprotease family, involved in Toxoplasma gondii egress from host cells and maturation of microneme proteins	TGME49_chrVIIa:903405-917753(+)	TGME49_206510	Toxoplasma_gondii_ME49	246741	10	2
Hypothetical protein	No function described	TGME49_chrVIIa:3523795-3527924(−)	TGME49_202200	Toxoplasma_gondii_ME49	87043	10	ND
Cytosolic tRNA-Ala synthetase	Cytosolic protein involved in the aminoacylation of tRNA during proteins synthesis	TGME49_chrXII:862344-876041(−)	TGME49_219540	Toxoplasma_gondii_ME49	139516	7	4
Vacuolar ATP synthase subunit A, putative	Proton-transporting ATP synthase activity contributing to endomembrane compartments acidification, organelle maturation, and intracellular pH regulation	TGME49_chrVIIb:4315661-4325274(−)	TGME49_256970	Toxoplasma_gondii_ME49	68122	6	3
Hypothetical protein	No function described	TGME49_chrVIII:2878595-2883425(−)	TGME49_233890	Toxoplasma_gondii_ME49	73697	6	2
Membrane Occupation and Recognition Nexus protein 2 (MORN2)	No function described, hypothetical role in cytoskeleton organization	TGME49_chrIX:4562922-4564319(+)	TGME49_292120	Toxoplasma_gondii_ME49	49236	6	ND
Hypothetical protein	No function described	TGME49_chrII:644118-650964(+)	TGME49_221870	Toxoplasma_gondii_ME49	144135	6	ND
Heat Shock Protein 90, putative	Molecular chaperone involved in protein folding, cellular stress responses, and regulation of host immune system	TGME49_chrVI:3356828-3362144(+)	TGME49_244560	Toxoplasma_gondii_ME49	96823	5	1
Aconitate hydratase ACN/IRP	Mitochondrial/apicoplast enzyme involved in the energy metabolism of Toxoplasma gondii (Krebs cycle) and regulation of host immune system (modulation of nitric oxide production)	TGME49_chrX:1400984-1408155(−)	TGME49_226730	Toxoplasma_gondii_ME49	114885	5	1
SAG-related sequence SRS29B (SRS29B)	Surface protein involved in host cell attachment	TGME49_chrVIII:2664481-2665491(+)	TGME49_233460	Toxoplasma_gondii_ME49	34732	4	4
DnaK family protein	ATP-dependent chaperone involved in protein folding	TGME49_chrX:1311834-1315968(−)	TGME49_226830	Toxoplasma_gondii_ME49	103193	4	2
Tetratricopeptide repeat-containing protein	ATP-dependent chaperone involved in protein folding	TGME49_chrIa:1252410-1260641(−)	TGME49_294898	Toxoplasma_gondii_ME49	88633	4	2
Actin 1 (ACT1)	Glideosome protein involved in gliding movement of Toxoplasma gondii, organelles distribution during cell division and secretory vesicles movement within the parasite	TGME49_chrIb:998462-1000024(+)	TGME49_209030	Toxoplasma_gondii_ME49	41908	3	3
Alpha Tubulin A1 (TUBA1)	Microtubule-associated protein involved in maintaining Toxoplasma gondii’s shape, intracellular transport, and apical complex organization	TGME49_chrXI:5274572-5276593(+)	TGME49_316400	Toxoplasma_gondii_ME49	50114	3	2
HEAT repeat-containing protein	Protein involved in intracellular transport, chromosome segregation, and signal transduction	TGME49_chrXI:6061226-6067819(−)	TGME49_216590	Toxoplasma_gondii_ME49	111196	3	ND
Hypothetical protein	No function described	TGME49_chrVIIa:2062064-2074174(+)	TGME49_203980	Toxoplasma_gondii_ME49	201280	3	ND
Chaperonin cpn60, putative	ATP-dependent chaperone involved in protein folding	TGME49_chrVI:1335457-1339036(+)	TGME49_240600	Toxoplasma_gondii_ME49	72237	3	ND
Protein disulfide isomerase	Endoplasmic reticulum enzyme involved in protein folding and redox homeostasis contributing to maturation and quality control of secretory and membrane proteins	TGME49_chrIV:1626551-1628375(+)	TGME49_211680	Toxoplasma_gondii_ME49	52802	2	3
Hypothetical protein	No function described	TGME49_chrXI:4609959-4617338(−)	TGME49_315270	Toxoplasma_gondii_ME49	84600	2	2
Histone lysine methyltransferase, SET, putative	Histone-lysine N-methyltransferase activity involved in epigenetic regulation	TGME49_chrVIIb:1042995-1044560(−)	TGME49_262750	Toxoplasma_gondii_ME49	53633	2	1
EMP24/GP25L/p24 family protein	Family of proteins involved in protein transport between endoplasmic reticulum, Golgi, and specialized organelles (micronemes or rhoptries)	TGME49_chrXI:1568699-1573498(+)	TGME49_310750	Toxoplasma_gondii_ME49	64977	2	1
D-3-phosphoglycerate dehydrogenase	Enzyme contributing to amino acid metabolism and providing precursors for nucleotide and lipid biosynthesis	TGME49_chrVI:939130-947919(−)	TGME49_239820	Toxoplasma_gondii_ME49	65194	2	ND
Cell division protein C48CY (CDC48CY)	AAA + ATPase protein involved in protein unfolding, degradation, membrane fusion, and regulation of turnover during cell cycle transitions	TGME49_chrVIII:3664539-3673736(−)	TGME49_273090	Toxoplasma_gondii_ME49	89397	2	ND
Microneme protein 1 (MIC1)	Protein secreted from apical micronemes during initial stages of host cell entry, forming a complex with MIC4 and MIC6 to mediate adhesion to host receptors	TGME49_chrIX:4395345-4397688(−)	TGME49_291890	Toxoplasma_gondii_ME49	48629	1	2
Inner Membrane Complex protein IMC2A (IMC2A)	Inner membrane complex protein contributing to the structural integrity and organization of the parasite pellicle	TGME49_chrX:520228-525722(−)	TGME49_228170	Toxoplasma_gondii_ME49	170816	ND	11
Cathepsin B	Cysteine protease localized to the endolysosomal system, involved in proteolytic processing, digestion of host macromolecules, and protein turnover	TGME49_chrXII:4721208-4726837(−)	TGME49_249670	Toxoplasma_gondii_ME49	62477	ND	8
Nuclear Factor 4 (NF4)	Putative transcription factor regulating gene expression	TGME49_chrVIIa:1293654-1297697(+)	TGME49_205580	Toxoplasma_gondii_ME49	51221	ND	7
Alveolin domain containing intermediate filament IMC4 (ALV4)	Inner membrane complex protein contributing to the cytoskeletal scaffold that provides mechanical strength and structural support to the parasite	TGME49_chrVIII:1513141-1515301(−)	TGME49_231630	Toxoplasma_gondii_ME49	50172	ND	4
Ubiquitin family protein	Regulator of protein turnover through the ubiquitin-proteasome system	TGME49_chrV:633282-639366(+)	TGME49_212820	Toxoplasma_gondii_ME49	119003	ND	3
Hypothetical protein	No function described	TGME49_chrXI:1880862-1900195(+)	TGME49_311230	Toxoplasma_gondii_ME49	504758	ND	2
AMP-binding enzyme domain-containing protein	Protein involved in metabolic pathways such as fatty acid activation, amino acid adenylation, and secondary metabolite biosynthesis, contributing to parasite energy metabolism and adaptation	TGME49_chrXI:1092557-1096957(−)	TGME49_310150	Toxoplasma_gondii_ME49	95937	ND	2
NTPase I	Enzyme involved in energy metabolism, signal transduction, and regulation of nucleotide hydrolysis-dependent cellular processes	TGME49_chrXII:6699033-6699642(−)	TGME49_277240	Toxoplasma_gondii_ME49	22641	ND	2
Dynamin-Related Protein B (DRPB)	Member of the dynamin superfamily of GTPases involved in membrane remodeling processes	TGME49_chrIb:1689989-1700172(+)	TGME49_321620	Toxoplasma_gondii_ME49	95931	ND	2

**Table 2:** Proteins of *Toxoplasma gondii* RH strain identified by LC-MS/MS for the second IgM triplet spot of interest (spot 22) (ND: not detected).

Protein name	Known or predicted function	Location	Accession	Organism	Molecular weight (Da)	Peptide count on protein profile No. 1	Peptide count on protein profile No. 2
Heat Shock Protein (HSP)	Molecular chaperone involved in protein folding, cellular stress responses, and regulation of host immune system	TGME49_chrXII:5582774-5587740(−)	TGME49_251780	Toxoplasma_gondii_ME49	78251	46	60
Heat Shock Protein 70 (HSP70)	Molecular chaperone involved in protein folding, cellular stress responses, and regulation of host immune system (modulation of nitric oxide production and reduction of macrophages phagocytosis capacity)	TGME49_chrVIII:3289396-3291420(−)	TGME49_273760	Toxoplasma_gondii_ME49	72880	43	42
Heat Shock Protein 90 (HSP90)	Molecular chaperone involved in protein folding, cellular stress responses, and regulation of host immune system	TGME49_chrIX:2483404-2487798(+)	TGME49_288380	Toxoplasma_gondii_ME49	81933	27	25
Chaperonin protein BiP	Molecular chaperone involved in protein folding and exit from the endoplasmic reticulum	TGME49_chrXI:2225378-2228753(−)	TGME49_311720	Toxoplasma_gondii_ME49	73253	26	40
Alveolin domain containing intermediate filament IMC1 (ALV1)	Inner membrane complex protein associated with the membrane cytoskeleton of T*oxoplasma gondii*, involved in maintaining parasite morphology and motility	TGME49_chrVIII:1516857-1519632(+)	TGME49_231640	Toxoplasma_gondii_ME49	69596	15	25
Cytosolic tRNA-Ala synthetase	Cytosolic protein involved in the aminoacylation of tRNA during proteins synthesis	TGME49_chrXII:862344-876041(−)	TGME49_219540	Toxoplasma_gondii_ME49	139516	6	7
Vacuolar ATP synthase subunit A, putative	Proton-transporting ATP synthase activity contributing to endomembrane compartments acidification, organelle maturation, and intracellular pH regulation	TGME49_chrVIIb:4315661-4325274(−)	TGME49_256970	Toxoplasma_gondii_ME49	68122	4	13
Protease inhibitor 1 (PI1)	Protein involved in modulation of protease activity by inhibiting cysteine or serine proteases	TGME49_chrXII:1832043-1834438(−)	TGME49_217430	Toxoplasma_gondii_ME49	47782	4	3
Nuclear Factor 4 (NF4)	Putative transcription factor regulating gene expression	TGME49_chrVIIa:1293654-1297697(+)	TGME49_205580	Toxoplasma_gondii_ME49	51221	4	ND
Hypothetical protein	No function described	TGME49_chrVIII:2878595-2883425(−)	TGME49_233890	Toxoplasma_gondii_ME49	73697	3	7
Toxolysin 4 (TLN4)	Member of the M16A metalloprotease family, involved in *Toxoplasma gondii* egress from host cells and maturation of microneme proteins	TGME49_chrVIIa:903405-917753(+)	TGME49_206510	Toxoplasma_gondii_ME49	246741	3	6
Tryptophanyl-tRNA synthetase (TrpRS2)	Apicoplast protein involved in the aminoacylation of tRNA during proteins synthesis	TGME49_chrIX:2473710-2478137(+)	TGME49_288360	Toxoplasma_gondii_ME49	77058	3	6
Lysine-tRNA ligase	Enzyme ensuring the fidelity of lysine incorporation into nascent polypeptides, playing a key role in protein synthesis and cellular function	TGME49_chrVIIa:1202972-1207953(+)	TGME49_205710	Toxoplasma_gondii_ME49	74395	3	ND
SAG-related sequence SRS29B (SRS29B)	Surface protein involved in host cell attachment	TGME49_chrVIII:2664481-2665491(+)	TGME49_233460	Toxoplasma_gondii_ME49	34732	2	2
Actin 1 (ACT1)	Glideosome protein involved in gliding movement of *Toxoplasma gondii*, organelles distribution during cell division and secretory vesicles movement within the parasite	TGME49_chrIb:998462-1000024(+)	TGME49_209030	Toxoplasma_gondii_ME49	41908	2	2
Hypothetical protein	No function described	TGME49_chrXI:4609959-4617338(−)	TGME49_315270	Toxoplasma_gondii_ME49	84600	2	2
Histone lysine methyltransferase, SET, putative	Histone-lysine N-methyltransferase activity involved in epigenetic regulation	TGME49_chrVIIb:1042995-1044560(−)	TGME49_262750	Toxoplasma_gondii_ME49	53633	2	1
Alveolin domain containing intermediate filament IMC4 (ALV4)	Inner membrane complex protein contributing to the cytoskeletal scaffold that provides mechanical strength and structural support to the parasite	TGME49_chrVIII:1513141-1515301(−)	TGME49_231630	Toxoplasma_gondii_ME49	50172	2	1
Ulp1 protease family, C-terminal catalytic domain-containing protein	Enzyme involved in the maturation of ubiquitin-like proteins such as SUMO, contributing to post-translational modification regulation	TGME49_chrIX:1711745-1725039(+)	TGME49_265190	Toxoplasma_gondii_ME49	329629	2	1
Inner Membrane Complex protein 2A (IMC2A)	Inner membrane complex protein contributing to the structural integrity and organization of the parasite pellicle	TGME49_chrX:520228-525722(−)	TGME49_228170	Toxoplasma_gondii_ME49	170816	2	1
EMP24/GP25L/p24 family protein	Family of proteins involved in protein transport between endoplasmic reticulum, Golgi, and specialized organelles (micronemes or rhoptries)	TGME49_chrXI:1568699-1573498(+)	TGME49_310750	Toxoplasma_gondii_ME49	64977	2	1
Chaperonin cpn60, putative	ATP-dependent chaperone involved in protein folding	TGME49_chrVI:1335457-1339036(+)	TGME49_240600	Toxoplasma_gondii_ME49	72237	ND	7
Peptidase M16 inactive domain-containing protein	Member of the M16A metalloprotease family, no function described	TGME49_chrX:716364-726488(+)	TGME49_227948	Toxoplasma_gondii_ME49	142764	ND	5
Hypothetical protein	No function described	TGME49_chrVIIa:3523795-3527924(−)	TGME49_202200	Toxoplasma_gondii_ME49	87043	ND	4
Membrane Occupation and Recognition Nexus protein 2 (MORN2)	No function described, hypothetical role in cytoskeleton organization	TGME49_chrIX:4562922-4564319(+)	TGME49_292120	Toxoplasma_gondii_ME49	49236	ND	4
Heat Shock Protein 90, putative	Molecular chaperone involved in protein folding, cellular stress responses, and regulation of host immune system	TGME49_chrVI:3356828-3362144(+)	TGME49_244560	Toxoplasma_gondii_ME49	96823	ND	2
Domain K- type RNA binding proteins family protein	Proteins involved in post-transcriptional regulation, including RNA stability, splicing, transport, and translation	TGME49_chrX:5186140-5192433(+)	TGME49_235930	Toxoplasma_gondii_ME49	64265	ND	2
Aconitate hydratase ACN/IRP	Mitochondrial/apicoplast enzyme involved in the energy metabolism of *Toxoplasma gondii* (Krebs cycle) and regulation of host immune system (modulation of nitric oxide production)	TGME49_chrX:1400984-1408155(−)	TGME49_226730	Toxoplasma_gondii_ME49	114885	ND	2
D-3-phosphoglycerate dehydrogenase	Enzyme contributing to amino acid metabolism and providing precursors for nucleotide and lipid biosynthesis	TGME49_chrVI:939130-947919(−)	TGME49_239820	Toxoplasma_gondii_ME49	65194	ND	2

**Table 3:** Proteins of *Toxoplasma gondii* RH strain identified by LC-MS/MS for the third IgM triplet spot of interest (spot 23) (ND: not detected).

Protein name	Known or predicted function	Location	Accession	Organism	Molecular weight (Da)	Peptide count on protein profile No. 1	Peptide count on protein profile No. 2
Heat Shock Protein (HSP)	Molecular chaperone involved in protein folding, cellular stress responses, and regulation of host immune system	TGME49_chrXII:5582774-5587740(−)	TGME49_251780	Toxoplasma_gondii_ME49	78251	55	61
Chaperonin protein BiP	Molecular chaperone involved in protein folding and exit from the endoplasmic reticulum	TGME49_chrXI:2225378-2228753(−)	TGME49_311720	Toxoplasma_gondii_ME49	73253	27	43
Heat Shock Protein 70 (HSP70)	Molecular chaperone involved in protein folding, cellular stress responses, and regulation of host immune system (modulation of nitric oxide production and reduction of macrophages phagocytosis capacity)	TGME49_chrVIII:3289396-3291420(−)	TGME49_273760	Toxoplasma_gondii_ME49	72880	24	30
Vacuolar ATP synthase subunit A, putative	Proton-transporting ATP synthase activity contributing to endomembrane compartments acidification, organelle maturation, and intracellular pH regulation	TGME49_chrVIIb:4315661-4325274(−)	TGME49_256970	Toxoplasma_gondii_ME49	68122	20	24
Alveolin domain containing intermediate filament IMC1 (ALV1)	Inner membrane complex protein associated with the membrane cytoskeleton of T*oxoplasma gondii*, involved in maintaining parasite morphology and motility	TGME49_chrVIII:1516857-1519632(+)	TGME49_231640	Toxoplasma_gondii_ME49	69596	17	21
Cytosolic tRNA-Ala synthetase	Cytosolic protein involved in the aminoacylation of tRNA during proteins synthesis	TGME49_chrXII:862344-876041(−)	TGME49_219540	Toxoplasma_gondii_ME49	139516	12	24
Heat Shock Protein 90 (HSP90)	Molecular chaperone involved in protein folding, cellular stress responses, and regulation of host immune system	TGME49_chrIX:2483404-2487798(+)	TGME49_288380	Toxoplasma_gondii_ME49	81933	11	20
Hypothetical protein	No function described	TGME49_chrVIII:2878595-2883425(−)	TGME49_233890	Toxoplasma_gondii_ME49	73697	9	13
Chaperonin cpn60, putative	ATP-dependent chaperone involved in protein folding	TGME49_chrVI:1335457-1339036(+)	TGME49_240600	Toxoplasma_gondii_ME49	72237	6	12
Tryptophanyl-tRNA synthetase (TrpRS2)	Apicoplast protein involved in the aminoacylation of tRNA during proteins synthesis	TGME49_chrIX:2473710-2478137(+)	TGME49_288360	Toxoplasma_gondii_ME49	77058	4	13
Aconitate hydratase ACN/IRP	Mitochondrial/apicoplast enzyme involved in the energy metabolism of *Toxoplasma gondii* (Krebs cycle) and regulation of host immune system (modulation of nitric oxide production)	TGME49_chrX:1400984-1408155(−)	TGME49_226730	Toxoplasma_gondii_ME49	114885	3	9
Actin 1 (ACT1)	Glideosome protein involved in gliding movement of *Toxoplasma gondii*, organelles distribution during cell division and secretory vesicles movement within the parasite	TGME49_chrIb:998462-1000024(+)	TGME49_209030	Toxoplasma_gondii_ME49	41908	2	5
SAG-related sequence SRS29B (SRS29B)	Surface protein involved in host cell attachment	TGME49_chrVIII:2664481-2665491(+)	TGME49_233460	Toxoplasma_gondii_ME49	34732	2	4
ATPase, AAA family protein	AAA + ATPase family protein involved in protein unfolding, degradation, membrane fusion, and regulation of turnover during cell cycle transitions	TGME49_chrX:4365489-4372954(−)	TGME49_234420	Toxoplasma_gondii_ME49	66807	2	3
Hypothetical protein	No function described	TGME49_chrXI:4609959-4617338(−)	TGME49_315270	Toxoplasma_gondii_ME49	84600	2	2
Cell Division protein C48CY (CDC48CY)	AAA + ATPase protein involved in protein unfolding, degradation, membrane fusion, and regulation of turnover during cell cycle transitions	TGME49_chrVIII:3664539-3673736(−)	TGME49_273090	Toxoplasma_gondii_ME49	89397	2	2
Alveolin domain containing intermediate filament IMC3 (ALV3)	Structural component of the inner membrane complex involved in maintaining parasite morphology and mechanical integrity, serves as a scaffold during daughter cell formation	TGME49_chrXI:6475497-6477113(+)	TGME49_216000	Toxoplasma_gondii_ME49	58000	2	1
EMP24/GP25L/p24 family protein	Family of proteins involved in protein transport between endoplasmic reticulum, Golgi, and specialized organelles (micronemes or rhoptries)	TGME49_chrXI:1568699-1573498(+)	TGME49_310750	Toxoplasma_gondii_ME49	64977	1	4
Protein disulfide isomerase	Endoplasmic reticulum enzyme involved in protein folding and redox homeostasis contributing to maturation and quality control of secretory and membrane proteins	TGME49_chrIV:1626551-1628375(+)	TGME49_211680	Toxoplasma_gondii_ME49	52802	1	3
Dynamin-Related Protein B (DRPB)	Member of the dynamin superfamily of GTPases involved in membrane remodeling processes	TGME49_chrIb:1689989-1700172(+)	TGME49_321620	Toxoplasma_gondii_ME49	95931	1	3
Microneme protein 1 (MIC1)	Protein secreted from apical micronemes during initial stages of host cell entry, forming a complex with MIC4 and MIC6 to mediate adhesion to host receptors	TGME49_chrIX:4395345-4397688(−)	TGME49_291890	Toxoplasma_gondii_ME49	48629	1	2
Toxolysin 4 (TLN4)	Member of the M16A metalloprotease family, involved in *Toxoplasma gondii* egress from host cells and maturation of microneme proteins	TGME49_chrVIIa:903405-917753(+)	TGME49_206510	Toxoplasma_gondii_ME49	246741	1	2
Hypothetical protein	No function described	TGME49_chrVIIa:3523795-3527924(−)	TGME49_202200	Toxoplasma_gondii_ME49	87043	ND	7
Peptidase M16 inactive domain-containing protein	Member of the M16A metalloprotease family, no function described	TGME49_chrX:6310793-6320102(−)	TGME49_214490	Toxoplasma_gondii_ME49	148868	ND	5
FUSE-Binding Protein 2 / KH-type splicing regulatory protein (FUBP2)	Protein involved in transcriptional regulation and post-transcriptional control, including mRNA splicing, stabilization, and localization	TGME49_chrXI:5969861-5973900(−)	TGME49_216670	Toxoplasma_gondii_ME49	100094	ND	4
D-3-phosphoglycerate dehydrogenase	Enzyme contributing to amino acid metabolism and providing precursors for nucleotide and lipid biosynthesis	TGME49_chrVI:939130-947919(−)	TGME49_239820	Toxoplasma_gondii_ME49	65194	ND	4
Hypothetical protein	No function described	TGME49_chrIII:1831237-1844046(−)	TGME49_254570	Toxoplasma_gondii_ME49	221185	ND	4
RNA recognition motif-containing protein	Protein involved in various aspects of RNA metabolism including splicing, transport, stability, and translation	TGME49_chrIX:4405615-4411375(+)	TGME49_291930	Toxoplasma_gondii_ME49	73118	ND	3
Domain K- type RNA binding proteins family protein	Proteins involved in post-transcriptional regulation, including RNA stability, splicing, transport, and translation	TGME49_chrX:5186140-5192433(+)	TGME49_235930	Toxoplasma_gondii_ME49	64265	ND	2
Apical Membrane Antigen 1 (AMA1)	Microneme protein secreted onto the parasite surface during host cell invasion, plays a key role in moving junction formation by interacting with rhoptry neck proteins	TGME49_chrVIIb:4948067-4952962(+)	TGME49_255260	Toxoplasma_gondii_ME49	63021	ND	2
Peptidase M16 inactive domain-containing protein	Member of the M16A metalloprotease family, no function described	TGME49_chrX:716364-726488(+)	TGME49_227948	Toxoplasma_gondii_ME49	142764	ND	2
Membrane Occupation and Recognition Nexus protein 2 (MORN2)	No function described, hypothetical role in cytoskeleton organization	TGME49_chrIX:4562922-4564319(+)	TGME49_292120	Toxoplasma_gondii_ME49	49236	ND	2
Hypothetical protein	No function described	TGME49_chrII:644118-650964(+)	TGME49_221870	Toxoplasma_gondii_ME49	144135	ND	2

**Table 4:** Proteins of *Toxoplasma gondii* RH strain identified by LC-MS/MS for the fourth IgM triplet spot of interest (spot 24) (ND: not detected).

Protein name	Known or predicted function	Location	Accession	Organism	Molecular weight (Da)	Peptide count on protein profile No. 1	Peptide count on protein profile No. 2
Heat Shock Protein (HSP)	Molecular chaperone involved in protein folding, cellular stress responses, and regulation of host immune system	TGME49_chrXII:5582774-5587740(−)	TGME49_251780	Toxoplasma_gondii_ME49	78251	51	45
Chaperonin protein BiP	Molecular chaperone involved in protein folding and exit from the endoplasmic reticulum	TGME49_chrXI:2225378-2228753(−)	TGME49_311720	Toxoplasma_gondii_ME49	73253	33	30
Cytosolic tRNA-Ala synthetase	Cytosolic protein involved in the aminoacylation of tRNA during proteins synthesis	TGME49_chrXII:862344-876041(−)	TGME49_219540	Toxoplasma_gondii_ME49	139516	29	21
Vacuolar ATP synthase subunit A, putative	Proton-transporting ATP synthase activity contributing to endomembrane compartments acidification, organelle maturation, and intracellular pH regulation	TGME49_chrVIIb:4315661-4325274(−)	TGME49_256970	Toxoplasma_gondii_ME49	68122	28	17
Tryptophanyl-tRNA synthetase (TrpRS2)	Apicoplast protein involved in the aminoacylation of tRNA during proteins synthesis	TGME49_chrIX:2473710-2478137(+)	TGME49_288360	Toxoplasma_gondii_ME49	77058	24	10
Chaperonin cpn60, putative	ATP-dependent chaperone involved in protein folding	TGME49_chrVI:1335457-1339036(+)	TGME49_240600	Toxoplasma_gondii_ME49	72237	23	11
Alveolin domain containing intermediate filament IMC1 (ALV1)	Inner membrane complex protein associated with the membrane cytoskeleton of T*oxoplasma gondii*, involved in maintaining parasite morphology and motility	TGME49_chrVIII:1516857-1519632(+)	TGME49_231640	Toxoplasma_gondii_ME49	69596	14	12
Heat Shock Protein 70 (HSP70)	Molecular chaperone involved in protein folding, cellular stress responses, and regulation of host immune system (modulation of nitric oxide production and reduction of macrophages phagocytosis capacity)	TGME49_chrVIII:3289396-3291420(−)	TGME49_273760	Toxoplasma_gondii_ME49	72880	13	12
Hypothetical protein	No function described	TGME49_chrVIII:2878595-2883425(−)	TGME49_233890	Toxoplasma_gondii_ME49	73697	11	7
Aconitate hydratase ACN/IRP	Mitochondrial/apicoplast enzyme involved in the energy metabolism of *Toxoplasma gondii* (Krebs cycle) and regulation of host immune system (modulation of nitric oxide production)	TGME49_chrX:1400984-1408155(−)	TGME49_226730	Toxoplasma_gondii_ME49	114885	10	3
Cell Division protein C48CY (CDC48CY)	AAA + ATPase protein involved in protein unfolding, degradation, membrane fusion, and regulation of turnover during cell cycle transitions	TGME49_chrVIII:3664539-3673736(−)	TGME49_273090	Toxoplasma_gondii_ME49	89397	7	4
FUSE-Binding Protein 2 / KH-type splicing regulatory protein (FUBP2)	Protein involved in transcriptional regulation and post-transcriptional control, including mRNA splicing, stabilization, and localization	TGME49_chrXI:5969861-5973900(−)	TGME49_216670	Toxoplasma_gondii_ME49	100094	7	1
Toxolysin 4 (TLN4)	Member of the M16A metalloprotease family, involved in *Toxoplasma gondii* egress from host cells and maturation of microneme proteins	TGME49_chrVIIa:903405-917753(+)	TGME49_206510	Toxoplasma_gondii_ME49	246741	6	ND
ATPase, AAA family protein	AAA + ATPase family protein involved in protein unfolding, degradation, membrane fusion, and regulation of turnover during cell cycle transitions	TGME49_chrX:4365489-4372954(−)	TGME49_234420	Toxoplasma_gondii_ME49	66807	5	1
RNA recognition motif-containing protein	Protein involved in various aspects of RNA metabolism including splicing, transport, stability, and translation	TGME49_chrIX:4405615-4411375(+)	TGME49_291930	Toxoplasma_gondii_ME49	73118	5	1
SAG-related sequence SRS29B (SRS29B)	Surface protein involved in host cell attachment	TGME49_chrVIII:2664481-2665491(+)	TGME49_233460	Toxoplasma_gondii_ME49	34732	4	2
Actin 1 (ACT1)	Glideosome protein involved in gliding movement of *Toxoplasma gondii*, organelles distribution during cell division and secretory vesicles movement within the parasite	TGME49_chrIb:998462-1000024(+)	TGME49_209030	Toxoplasma_gondii_ME49	41908	4	2
Heat Shock Protein 90 (HSP90)	Molecular chaperone involved in protein folding, cellular stress responses, and regulation of host immune system	TGME49_chrIX:2483404-2487798(+)	TGME49_288380	Toxoplasma_gondii_ME49	81933	4	1
D-3-phosphoglycerate dehydrogenase	Enzyme contributing to amino acid metabolism and providing precursors for nucleotide and lipid biosynthesis	TGME49_chrVI:939130-947919(−)	TGME49_239820	Toxoplasma_gondii_ME49	65194	4	1
Dynamin-Related Protein B (DRPB)	Member of the dynamin superfamily of GTPases involved in membrane remodeling processes	TGME49_chrIb:1689989-1700172(+)	TGME49_321620	Toxoplasma_gondii_ME49	95931	4	1
Microneme protein 1 (MIC1)	Protein secreted from apical micronemes during initial stages of host cell entry, forming a complex with MIC4 and MIC6 to mediate adhesion to host receptors	TGME49_chrIX:4395345-4397688(−)	TGME49_291890	Toxoplasma_gondii_ME49	48629	4	ND
EMP24/GP25L/p24 family protein	Family of proteins involved in protein transport between endoplasmic reticulum, Golgi, and specialized organelles (micronemes or rhoptries)	TGME49_chrXI:1568699-1573498(+)	TGME49_310750	Toxoplasma_gondii_ME49	64977	3	1
Hypothetical protein	No function described	TGME49_chrVIIa:3523795-3527924(−)	TGME49_202200	Toxoplasma_gondii_ME49	87043	3	ND
Protein disulfide isomerase	Endoplasmic reticulum enzyme involved in protein folding and redox homeostasis contributing to maturation and quality control of secretory and membrane proteins	TGME49_chrIV:1626551-1628375(+)	TGME49_211680	Toxoplasma_gondii_ME49	52802	2	1
Heat Shock Protein 90, putative	Molecular chaperone involved in protein folding, cellular stress responses, and regulation of host immune system	TGME49_chrVI:3356828-3362144(+)	TGME49_244560	Toxoplasma_gondii_ME49	96823	2	ND
Ubiquitin specific protease 39 isoform 2, putative	Protease involved in spliceosome assembly, regulation of pre-mRNA splicing and proper gene expression	TGME49_chrIa:840906-848857(+)	TGME49_294360	Toxoplasma_gondii_ME49	64190	2	ND
Domain K- type RNA binding proteins family protein	Proteins involved in post-transcriptional regulation, including RNA stability, splicing, transport, and translation	TGME49_chrX:5186140-5192433(+)	TGME49_235930	Toxoplasma_gondii_ME49	64265	2	ND
Apical Membrane Antigen 1 (AMA1)	Microneme protein secreted onto the parasite surface during host cell invasion, plays a key role in moving junction formation by interacting with rhoptry neck proteins	TGME49_chrVIIb:4948067-4952962(+)	TGME49_255260	Toxoplasma_gondii_ME49	63021	2	ND
Alpha Tubulin A1 (TUBA1)	Microtubule-associated protein involved in maintaining *Toxoplasma gondii’s* shape, intracellular transport, and apical complex organization	TGME49_chrXI:5274572-5276593(+)	TGME49_316400	Toxoplasma_gondii_ME49	50114	2	ND
Hypothetical protein	No function described	TGME49_chrXI:4609959-4617338(−)	TGME49_315270	Toxoplasma_gondii_ME49	84600	2	ND

Some proteins were only detected on one of the two silver-stained protein profiles of the *T. gondii* RH strain and were therefore excluded. As a reminder, the IgM triplet is made up of three high molecular weight (MW) bands of 75, 90, and 100 kDa. Proteins identified for each spot (21–24) were grouped according to their MW, to match the three IgM triplet bands (75, 90, and 100 kDa) as closely as possible (Table 5). Low MW proteins (less than 65 kDa) were excluded, as the first IgM triplet band appeared at 75 kDa. On the other hand, high MW proteins (greater than 100 kDa) were associated with the last IgM triplet band at 100 kDa, as resolution is limited in this region, making it impossible to exclude them.

**Table 5:** Proteins identified by LC-MS/MS for spots 21–24 classified according to their molecular weight and assigned to the IgM triplet band with the closest molecular weight. Proteins with too low molecular weight (below 65 kDa) were not considered to belong to the IgM triplet. High molecular weight proteins (over 100 kDa) were integrated into the IgM triplet, as protein resolution is lower in this zone (ND: not detected). Lines in bold refer to the thirteen main candidates for the IgM triplet.

	Protein name for spot No. 21	Protein name for spot No. 22	Protein name for spot No. 23	Protein name for spot No. 24
Low molecular weight	SAG-related sequence SRS29B (SRS29B)	SAG-related sequence SRS29B (SRS29B)	SAG-related sequence SRS29B (SRS29B)	SAG-related sequence SRS29B (SRS29B)
	Actin 1 (ACT1)	Actin 1 (ACT1)	Actin 1 (ACT1)	Actin 1 (ACT1)
	ND	Protease Inhibitor 1 (PI1)	ND	ND
	Microneme protein 1 (MIC1)	ND	Microneme protein 1 (MIC1)	ND
	Alpha Tubulin A1 (TUBA1)	ND	ND	ND
	ND	Alveolin domain containing intermediate filament IMC4 (ALV4)	ND	ND
	Protein disulfide isomerase	ND	Protein disulfide isomerase	Protein disulfide isomerase
	Histone lysine methyltransferase, SET, putative	Histone lysine methyltransferase, SET, putative	ND	ND
	ND	ND	Alveolin domain containing intermediate filament IMC3 (ALV3)	ND
75 kDa band	**EMP24/GP25L/p24 family protein**	**EMP24/GP25L/p24 family protein**	**EMP24/GP25L/p24 family protein**	**EMP24/GP25L/p24 family protein**
	ND	ND	ND	D-3-phosphoglycerate dehydrogenase
	ND	ND	ATPase, AAA family protein	ATPase, AAA family protein
	**Vacuolar ATP synthase subunit A, putative**	**Vacuolar ATP synthase subunit A, putative**	**Vacuolar ATP synthase subunit A, putative**	**Vacuolar ATP synthase subunit A, putative**
	**Alveolin domain containing intermediate filament IMC1 (ALV1)**	**Alveolin domain containing intermediate filament IMC1 (ALV1)**	**Alveolin domain containing intermediate filament IMC1 (ALV1)**	**Alveolin domain containing intermediate filament IMC1 (ALV1)**
	ND	ND	Chaperonin cpn60, putative	Chaperonin cpn60, putative
	**Heat Shock Protein 70 (HSP70)**	**Heat Shock Protein 70 (HSP70)**	**Heat Shock Protein 70 (HSP70)**	**Heat Shock Protein 70 (HSP70)**
	ND	ND	ND	RNA recognition motif-containing protein
	**Chaperonin protein BiP**	**Chaperonin protein BiP**	**Chaperonin protein BiP**	**Chaperonin protein BiP**
	**Hypothetical protein**	**Hypothetical protein**	**Hypothetical protein**	**Hypothetical protein**
	**Tryptophanyl-tRNA synthetase (TrpRS2)**	**Tryptophanyl-tRNA synthetase (TrpRS2)**	**Tryptophanyl-tRNA synthetase (TrpRS2)**	**Tryptophanyl-tRNA synthetase (TrpRS2)**
	**Heat Shock Protein (HSP)**	**Heat Shock Protein (HSP)**	**Heat Shock Protein (HSP)**	**Heat Shock Protein (HSP)**
90 KDa band	**Heat Shock Protein 90 (HSP90)**	**Heat Shock Protein 90 (HSP90)**	**Heat Shock Protein 90 (HSP90)**	**Heat Shock Protein 90 (HSP90)**
	**Hypothetical protein**	**Hypothetical protein**	**Hypothetical protein**	ND
	Tetratricopeptide repeat-containing protein	ND	ND	ND
	ND	ND	Cell Division protein C48CY (CDC48CY)	Cell Division protein C48CY (CDC48CY)
	ND	ND	Dynamin-Related Protein B (DRPB)	Dynamin-Related Protein B (DRPB)
	Heat Shock Protein 90, putative	ND	ND	ND
100 kDa band	ND	ND	ND	FUSE-Binding Protein 2 / KH-type splicing regulatory protein (FUBP2)
	DnaK family protein	ND	ND	ND
	**Aconitate hydratase ACN/IRP**	ND	Aconitate hydratase ACN/IRP	Aconitate hydratase ACN/IRP
	**Cytosolic tRNA-Ala synthetase**	**Cytosolic tRNA-Ala synthetase**	**Cytosolic tRNA-Ala synthetase**	**Cytosolic tRNA-Ala synthetase**
	Peptidase M16 inactive domain-containing protein	ND	ND	ND
	ND	Inner Membrane Complex protein 2A (IMC2A)	ND	ND
	**Toxolysin 4 (TLN4)**	**Toxolysin 4 (TLN4)**	**Toxolysin 4 (TLN4)**	ND
	ND	Ulp1 protease family, C-terminal catalytic domain-containing protein	ND	ND

A total of eight proteins (EMP24/GP25L/p24 family protein; Vacuolar ATP synthase subunit A, putative; Alveolin domain containing intermediate filament IMC1 [ALV1]; Heat Shock Protein 70 [HSP70]; Chaperonin Protein BiP; hypothetical protein; Tryptophanyl-tRNA synthetase [TrpRS2]; and Heat Shock Protein family [HSP]) identified across the four selected spots were considered potential components of the 75 kDa IgM triplet band. Two proteins found in at least three of the four selected spots (Heat Shock Protein 90 [HSP90] and hypothetical protein) appeared to be good candidates for the 90 kDa IgM triplet band. Three proteins found in at least three of the four selected spots (Aconitate hydratase ACN/IRP; Cytosolic tRNA-Ala synthetase; and Toxolysin 4 [TLN4]) appeared to be good candidates for the 100 kDa IgM triplet band (Table 5).

## Discussion

The aim of this work was to identify the proteins involved in the immunogenic recognition marked by the IgM triplet, which is pathognomonic for CT [14]. This IgM triplet, represented by three high MW bands of 75, 90, and 100 kDa on the mother-infant IbPP, is thought to target proteins involved in maternal-fetal transmission of *T. gondii*. Identification of these proteins requires proteomic analysis.

Proteomics has evolved tremendously over the last few decades, thanks to technological and methodological advances that have notably made it possible to map most of the *T. gondii* proteome [4]. For example, in 2008, Xia *et al.* described almost one-third of the proteins in the tachyzoite stage of *T. gondii*, *i.e.*, approximately 2,252 proteins (including 2,477 intron-spanning peptides) using three different approaches (2-DE, LC-MS/MS, and multidimensional protein identification technology – MudPIT) [27]. In 2011, Che *et al.* similarly characterized 2,241 membrane proteins from the tachyzoite stage of *T. gondii* using one-dimensional gel electrophoresis LC-MS/MS, biotin labeling in conjunction with one-dimensional gel LC-MS/MS analysis, and a novel strategy that combines three-layer “sandwich” gel electrophoresis with multidimensional protein identification technology [3]. In 2017, Wang *et al.* identified by LC-MS/MS analysis some 6,285 proteins in *T. gondii* out of the 6,000 to 8,000 predicted by genomic analysis, these proteins being expressed differently depending on the parasite stage (tachyzoite, bradyzoite, or sporozoite) [3, 26]. All these advances in proteomic analysis have given rise to the *ToxoDB* database. The identification of these proteins provides a better understanding of their role in the various biological processes of the parasite cycle, as well as in the host-parasite interactions in which they are involved [4]. This actually makes it possible to characterize the proteins involved in the mechanisms used by *T. gondii* to invade, replicate, and manipulate the host’s metabolism, and establish its virulence. In fact, *T. gondii* invades host cells through a process known as gliding motility, which involves the coordinated action of proteins such as Myosin A, Actin filaments, Gliding-Associated Proteins (GAP), Microneme proteins, Rhoptry Neck proteins (RON), Apical Membrane Antigen 1 (AMA1), and Rhoptry proteins (ROPs).

These proteins work together to facilitate the attachment of toxoplasma to host cells, form a mobile junction, and ultimately invade and exit these host cells.

In our study, we sought to identify the proteins involved in the IgM triplet by 2-DE followed by LC-MS analysis.

Around the area of 75–100 kDa four spots of interest were found.

Several proteins are good candidates for the IgM triplet. These include eight proteins (EMP24/GP25L/p24 family protein; Vacuolar ATP synthase subunit A, putative; ALV1; HSP70; Chaperonin Protein BiP; hypothetical protein; TrpRS2, and HSP) for the 75 kDa band, two proteins (HSP90 and hypothetical protein) for the 90 kDa band, and three proteins (Aconitate Hydratase ACN/IRP; Cytosolic tRNA-Ala synthetase; and TLN4) for the 100 kDa band. It is unlikely that all thirteen identified proteins contribute to the immunological response underlying the IgM triplet. The IgM triplet is composed of three narrow and well-defined high MW bands, suggesting that only a few of the thirteen proteins are truly implicated, perhaps one or two proteins per band. The remaining proteins are likely co-migrating entities, either due to similar native molecular weights or the presence of post-translational modifications. The higher the abundance of protein within the parasite, the more likely it is to be recognized by the host immune system. According to the ToxoDB database (ToxoDB-10.0_TgondiiME49_AnnotatedProteins.fasta, 8,322 sequences), transcript levels exceed 1,000 transcripts per million (TPM) for proteins such as HSP70, HSP90, and ALV1, suggesting that these proteins are strong candidates as true targets of the IgM triplet. In contrast, other proteins identified, such as TLN4, exhibit TPM values well below 1,000, which argues against their significant involvement in IgM triplet formation. Additional studies will be required to identify which of these proteins represent the real targets of the IgM triplet.

It turns out that each of these proteins is involved in some way in the cell invasion process, and therefore probably in the transplacental invasion process of *T. gondii*.

For the 75 kDa and 90 kDa IgM triplet bands, HSP family predominate. More specifically, HSP70 and HSP90 were found, with respective MWs of 73 and 82 kDa. In a previous work, we showed that these proteins are known to be essential for *T. gondii* host cell invasion and virulence [2]. For the 75 kDa band, HSP70 is particularly well characterized in protozoa, and is also capable of interfering with its host’s immune system by modulating nitric oxide production by macrophages and reducing their phagocytosis capacity [2]. Lyons and Johnson have shown that the expression of HSP70 induced by immunological stress in *T. gondii* can protect it from the cellular damage associated with host invasion [16].

Another major protein for the 75 kDa band of the IgM triplet is the Chaperonin Protein BiP with a recorded MW of 73 kDa. It is a protein present in the endoplasmic reticulum (ER) of toxoplasma that may play a role in parasite invasion *via* its action on the secretory pathway. It is currently known to act as a molecular chaperone in protein folding and exit from the ER [2, 9]. For the 75 kDa band, there are four other proteins: Vacuolar ATP synthase subunit A, putative, 68 kDa; ALV1, 70 kDa; TrpRS2, 77 kDa; and EMP24/GP25L/p24 family protein, 65 kDa. V-ATPase subunit A is a catalytic subunit of the V-ATPase complex capable of pumping protons across membranes using the energy released by ATP hydrolysis. Like HSP proteins, this complex is synthesized in response to ionic stress to protect the toxoplasma from the host immune response. This pump also plays a role in the maturation of secretory proteins in endosomal compartments, such as microneme and rhoptry proteins [24].

ALV1 is a protein of the *T. gondii* inner membrane complex that has been little studied to date. It is associated with the membrane cytoskeleton of toxoplasma and therefore plays a role in the parasite’s motility and virulence [6, 7]. TrpRS2 is an enzyme located in the apicoplast of *T. gondii*, whose main function is the aminoacylation of tRNA during proteins synthesis [11]. The EMP24/GP25L/p24 protein family is a family of proteins that plays an important role in protein transport between the ER and the Golgi apparatus and specialized organelles such as micronemes or rhoptries in *T. gondii* [5]*.*

To finish with the 75 kDa band, another 74 kDa protein could also be involved, although it is less representative. It has been named “hypothetical protein” because it has not yet been characterized.

For the 90 kDa band, HSP90 plays a major role. Sun *et al.* showed that HSP90 knockout strains of *T. gondii* had a 57.5% decreased invasion capacity 1 hour after infection of Vero cells, and a 58.1% decreased intracellular growth at 24 h [25]. These are highly conserved chaperone proteins present in the mitochondria of toxoplasma and produced in response to the stress generated in the host by parasitic infection. They are involved in the correct folding of proteins, the degradation of damaged proteins and their transport across the mitochondrial membrane [2, 12, 25]. Another 85 kDa protein may also be involved, although it is less representative than HSP90. As with the 75 kDa band, it has been named “hypothetical protein”, as it has not yet been characterized.

For the 100 kDa band, three proteins stand out: Aconitate hydratase ACN/IRP (aconitase) with a MW of 115 kDa; Cytosolic tRNA-Ala synthetase with a MW of 140 kDa; and TLN4 with a MW of 247 kDa. The first protein mentioned is aconitase, a mitochondrial enzyme also presents in the apicoplast of toxoplasma. It plays an important role in the energy metabolism of *T. gondii*, essential to the parasite invasion process, thanks to its role in the Krebs cycle. In the study by MacRae *et al.*, inhibition of aconitase considerably reduced the sliding motility of toxoplasma [17].

It also plays a role in regulating the parasite’s iron metabolism. Aconitase thus plays a key role in maintaining cellular homeostasis in *T. gondii*. Like HSP70, it is capable of interfering with the host immune system by regulating the production of reactive oxygen species that are harmful to the parasite [22]. The second most widely represented protein is Cytosolic tRNA-Ala synthetase. Like TrpRS2, the primary function of Cytosolic tRNA-Ala synthetase is aminoacylation of tRNA, an essential step in protein synthesis. It also helps regulate protein synthesis by hydrolyzing misacylated amino acids [8]. Finally, the third protein is TLN4, a member of the M16A metalloprotease family. It plays a crucial role in the exit of the parasite from the host cell, allowing it to continue its lytic cycle by infecting other cells [2, 10]. Proteins secreted by apical micronemes are central components for host cell recognition, invasion, exit, and virulence. TLN4 may act as a maturase or regulator for these microneme proteins. TLN4 is first synthesized as a large precursor (~260 kDa) and then cleaved into several proteolytic fragments within the parasite’s secretory system [15].

These proteins are closely linked and work together to promote parasite invasion, either by acting as transporters for certain proteins in the secretory pathway, by regulating their maturation, or by modulating the host immune system to promote toxoplasma virulence. These processes are necessary prerequisites for the parasite to cross epithelial and immunological barriers, including those of the placenta [2, 23]. While our study does not establish a direct mechanistic role for these proteins in vertical transmission, these proteins represent strong candidates for futures studies about the placental invasion process with a new therapeutic perspective.

Importantly, there is a clear need to develop treatments aimed at blocking transplacental passage of *T. gondii*, thus preventing exposure of the fetus [18].

In terms of diagnostic prospects, IgM triplet immunoblot testing constitutes a pathognomonic marker of congenital toxoplasmosis, conferring high specificity for the diagnosis of congenital infection in neonates. However, the IgM triplet may be absent in an infected newborn who lacks IgM but shows IgG neosynthesis. So, developing a diagnostic test only based on IgM triplet detection isn’t essential. Nonetheless, it is important to ensure that this triplet is present in all diagnostic tests for congenital toxoplasmosis.

## Conclusion

The mechanisms underlying transplacental transmission of *Toxoplasma gondii* from an infected mother to her fetus are complex and not yet fully elucidated. Many proteins, secreted or not by toxoplasma for cellular invasion, could be involved in this process. It turns out that the IgM triplet, a pathognomonic marker of CT identified by L’Ollivier *et al.* and Peyclit *et al.* in mother-infant IbPP, appears to specifically target several of these proteins. This study allowed us to identify 13 proteins potentially targeted by the IgM triplet, all parts of the effector arsenal deployed by *T. gondii*. Further research is needed to confirm the precise roles of these proteins, with the ultimate goal of improving neonatal diagnosis and the clinical management of CT.

## Data Availability

All data are available within the article.

## Supplementary materials

*Supplementary data S1*: Example of an IgM and IgG mother and child immunoblot pair profile with identical profiles and the infant’s IgM triplet (pink box). The child was proved to be infected subsequently during follow-up. m, mother; i, infant. Reproduced from Peyclit *et al.* 2023 [21]. *Supplementary data S2*: Immunoblot obtained using sera positive for the IgM triplet (NNTPos; Patients 1BB to 16BB) or negative for this same IgM triplet (NNTNeg; Patients 17BB to 19BB). The black box indicates the location of spots 21 to 24 only found on NNTPos sera and marked by arrows when present. The immunoblot obtained with the NNTNeg sera from the patient 19BB shows spots 25 and 26, which were excluded because they were not specific to the IgM triplet. The supplementary material of this article is available at https://www.parasite-journal.org/10.1051/parasite/2025065/olm.
