# Supplementary material for: Protein characterization of the IgM triplet involved in the diagnosis of congenital toxoplasmosis
Source: Parasite. 2025 Nov 26;32:72. doi: 10.1051/parasite/2025065 (PMC12656376; doi:10.1051/parasite/2025065)
Supplement: Supplementary file 1 — Supplementary data S1: Example of an IgM and IgG mother and child immunoblot pair profile with identical profiles and the infant’s IgM triplet (pink box). The child was proved to be infected subsequently during follow-up. m, mother; i, infant. Reproduced from Peyclit et al. 2023 [21]. [file parasite-32-72-s1.pdf]

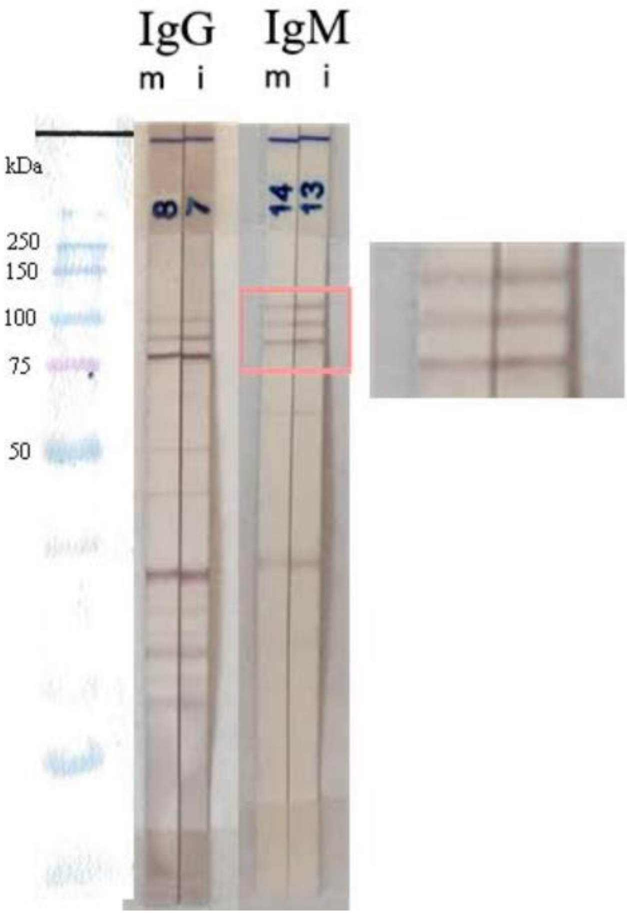

Supplementary data S1. Example of an IgM and IgG mother and child immunoblot pair profile with identical profiles and the infant's IgM triplet (pink box). The child was proved to be infected subsequently during follow-up. m, mother; i, infant. Reproduced from Peyclit L, Villard O, Paris L, Fricker-Hidalgo H, Houzé S, Cimon B, Deleplancque A-S, Tournus C, Pelloux H, Villena I, Pomares C, L'Ollivier C. 2023. IgM triplet in neonatal diagnosis by immunoblotting and its potential use as a diagnostic marker for congenital toxoplasmosis. Parasite; 30: 19.
