# Supplementary material for: Protein characterization of the IgM triplet involved in the diagnosis of congenital toxoplasmosis
Source: Parasite. 2025 Nov 26;32:72. doi: 10.1051/parasite/2025065 (PMC12656376; doi:10.1051/parasite/2025065)
Supplement: Supplementary file 2 — Supplementary data S2: Immunoblot obtained using sera positive for the IgM triplet (NNTPos; Patients 1BB to 16BB) or negative for this same IgM triplet (NNTNeg; Patients 17BB to 19BB). The black box indicates the location of spots 21 to 24 only found on NNTPos sera and marked by arrows when present. The immunoblot obtained with the NNTNeg sera from the patient 19BB shows spots 25 and 26, which were excluded because they were not specific to the IgM triplet. [file parasite-32-72-s2.pdf]

Patient 1BB

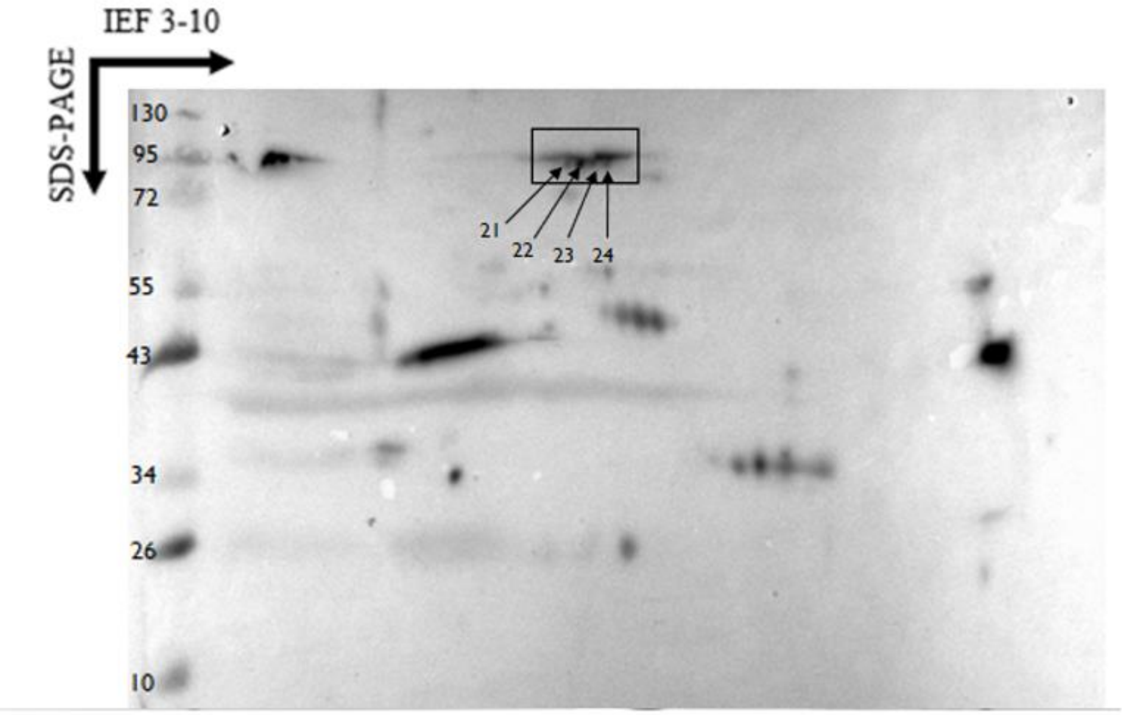

Patient 2BB

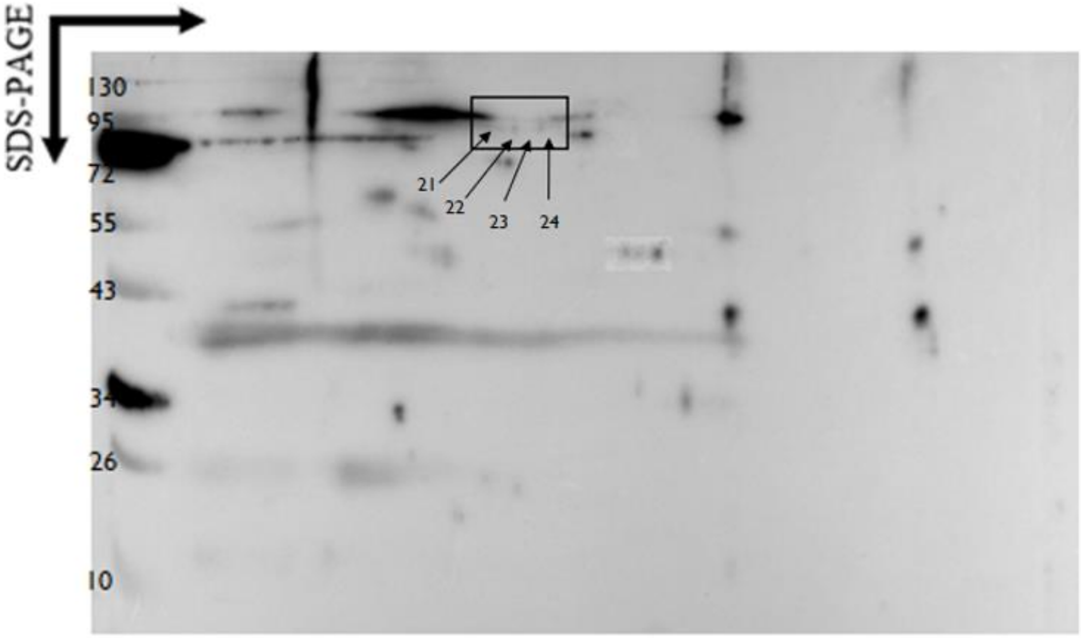

Patient 3BB

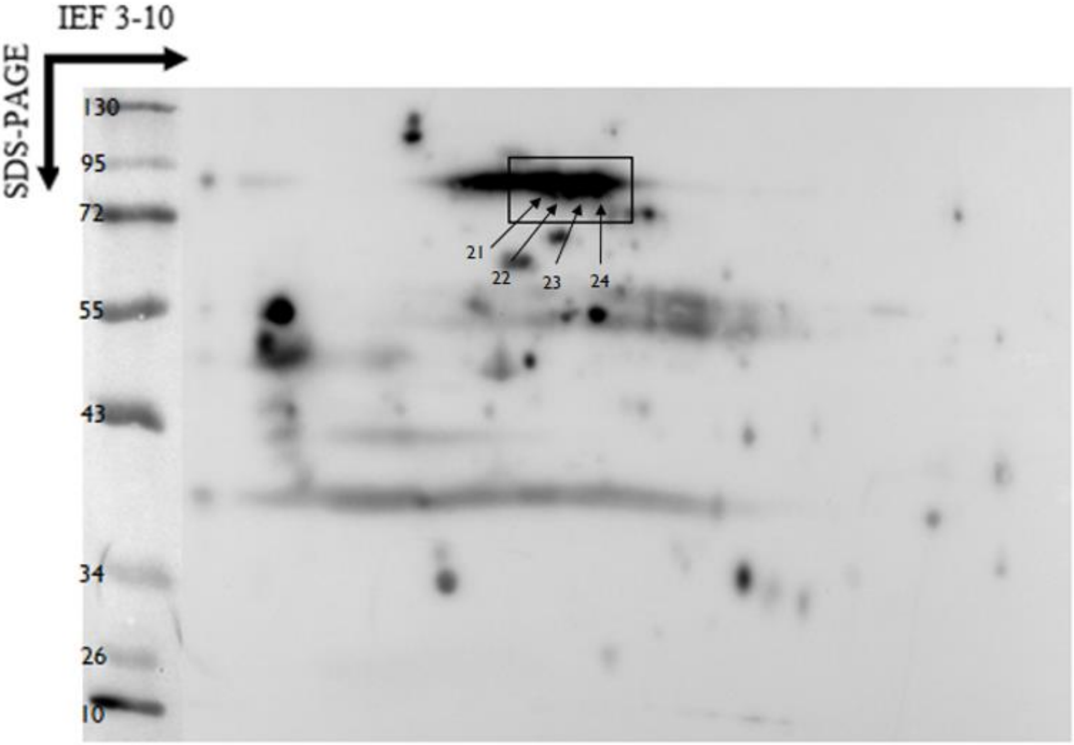

Patient 4BB

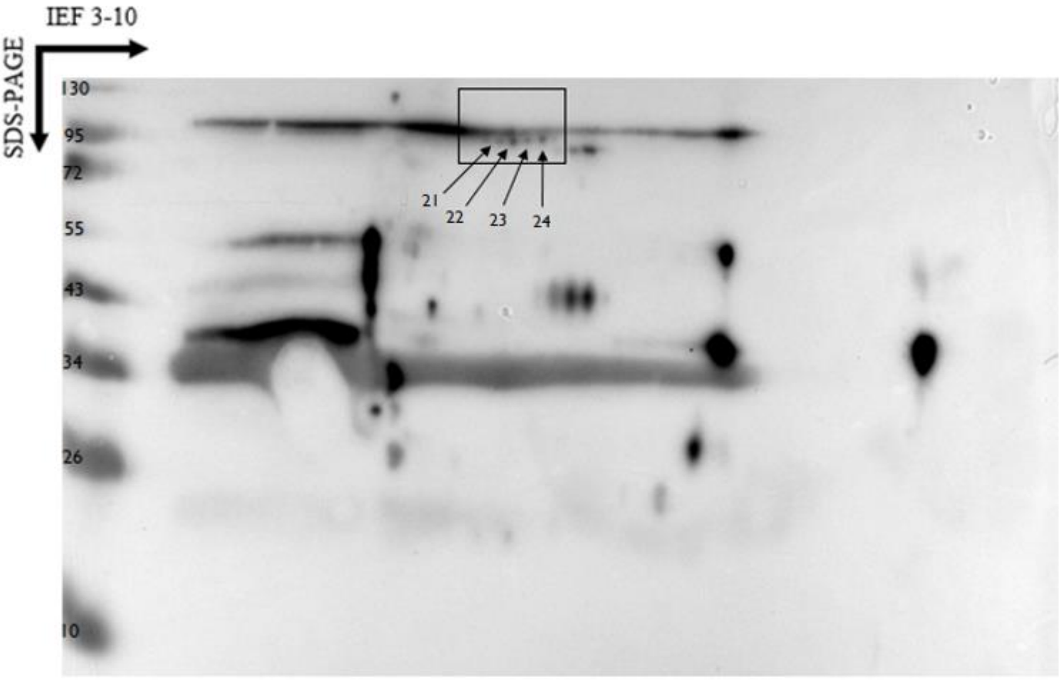

Patient 5BB

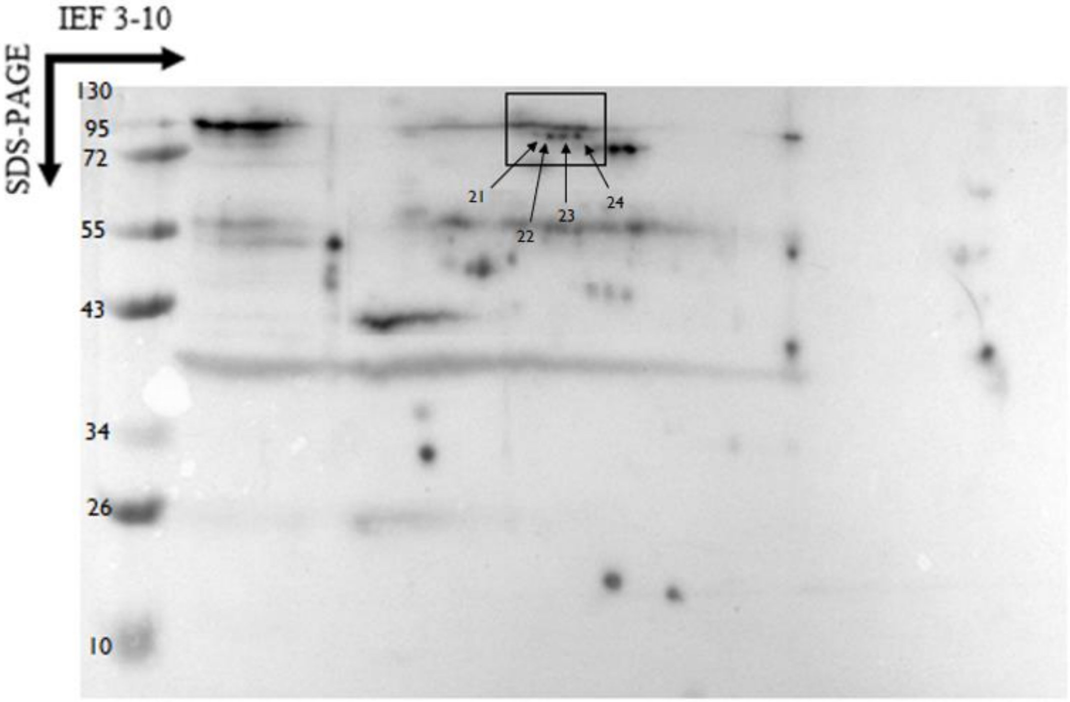

Patient 6BB

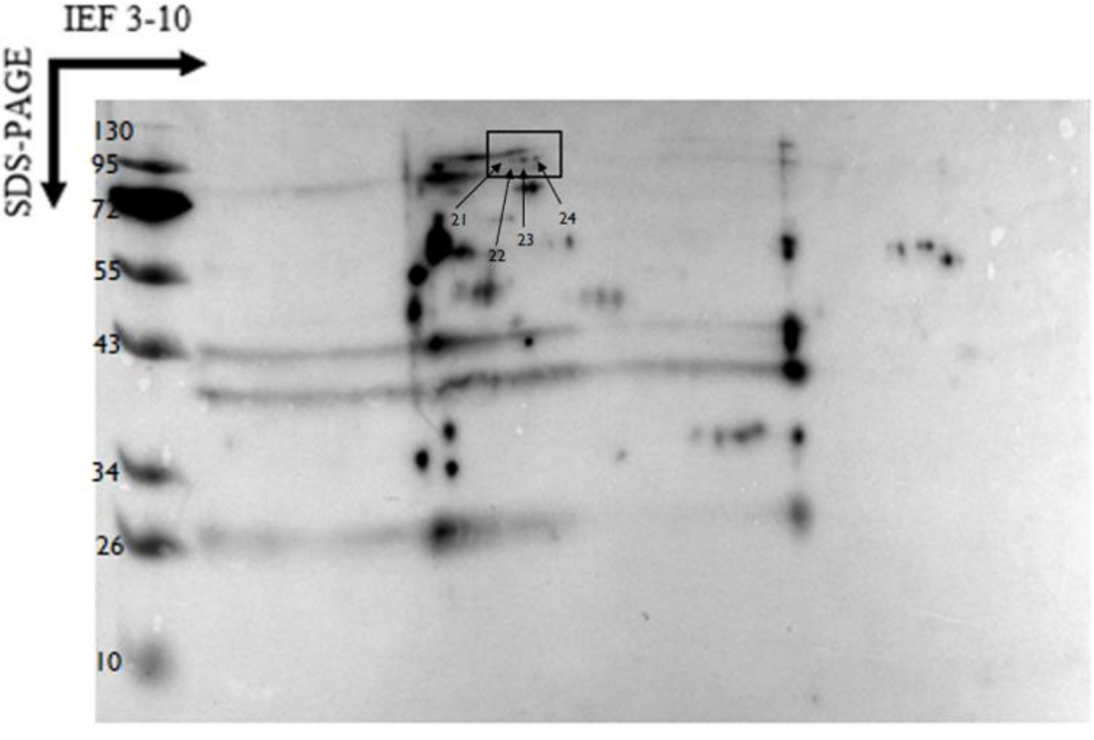

Patient 7BB

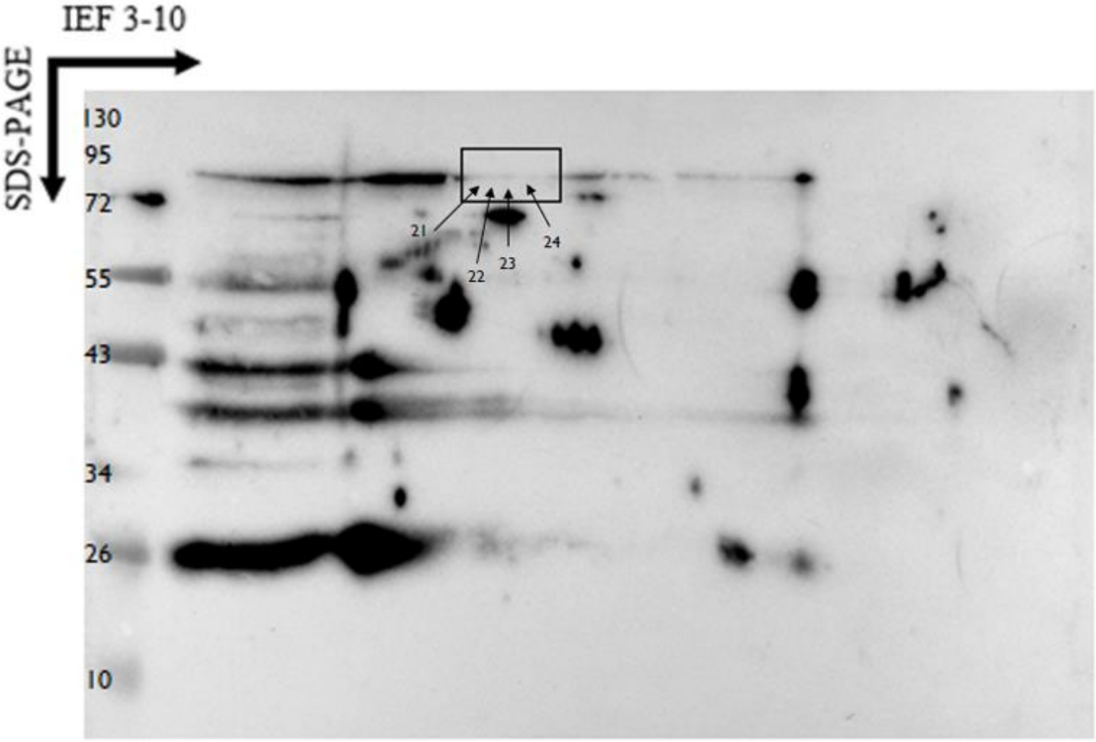

Patient 8BB

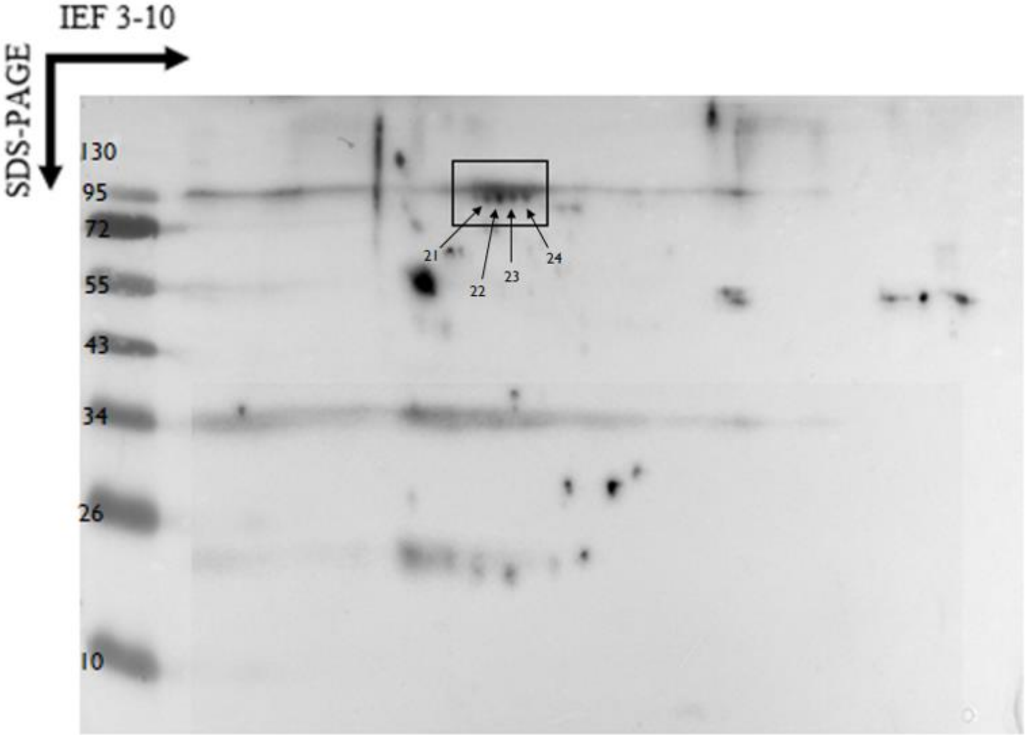

Patient 9BB
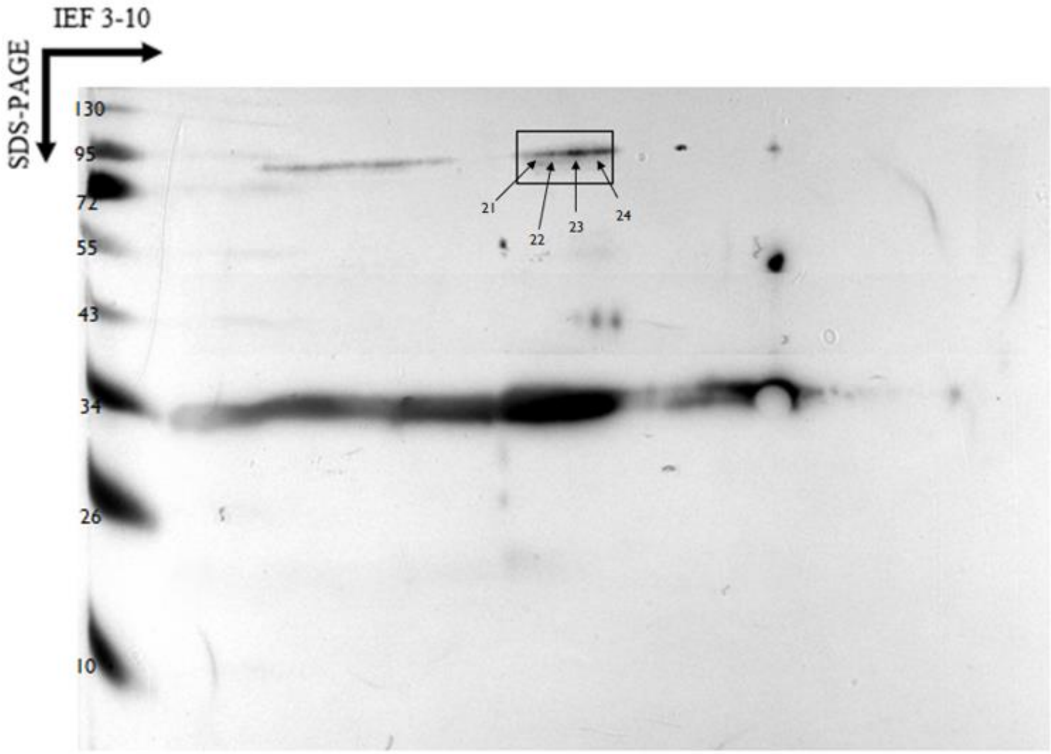
Patient 10BB
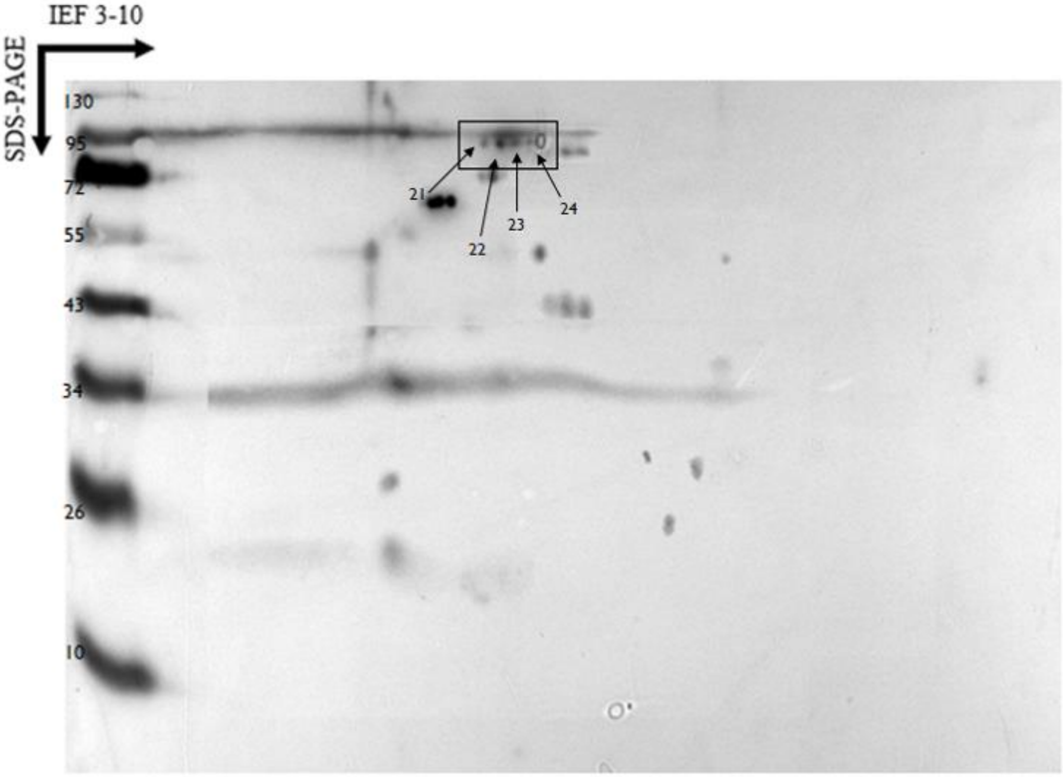

Patient 11BB
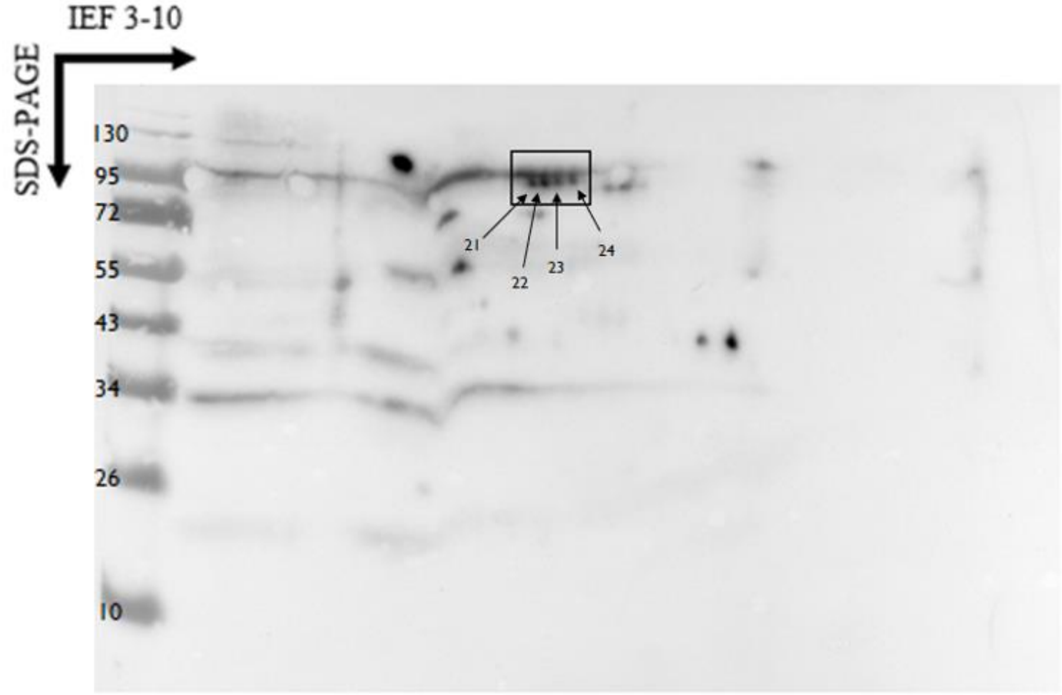
Patient 12BB
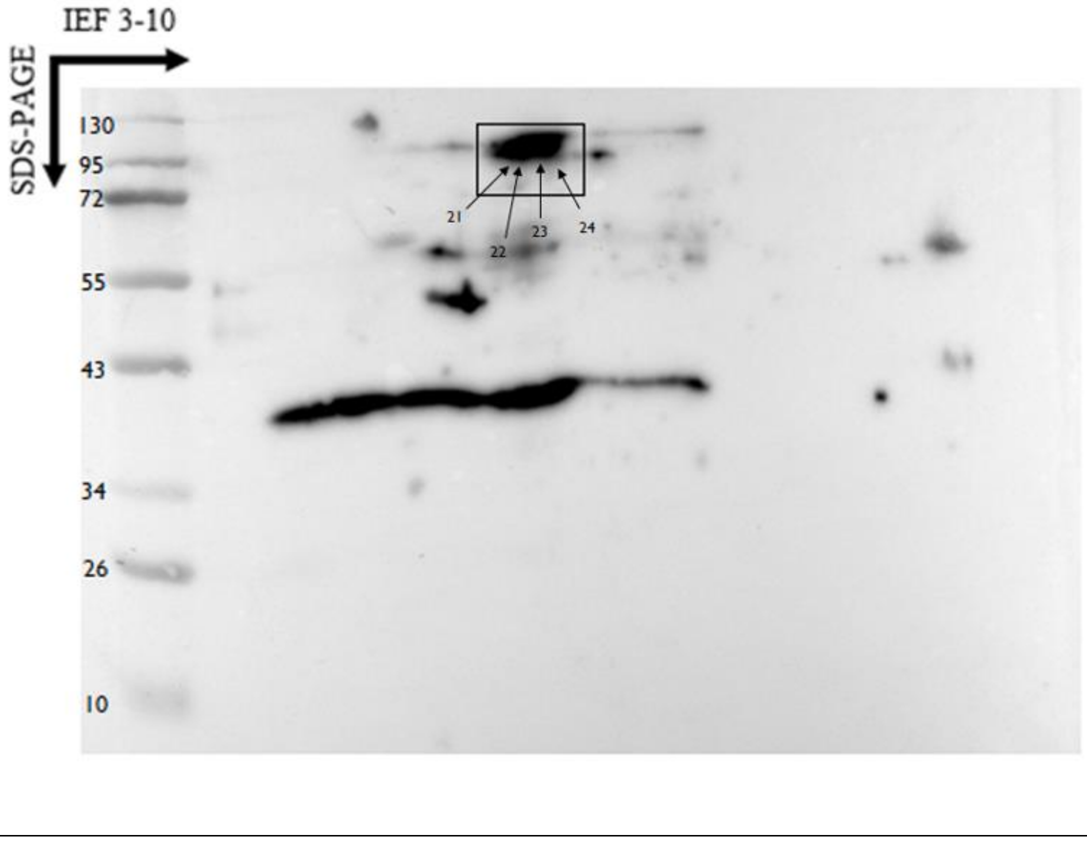

Patient 13BB
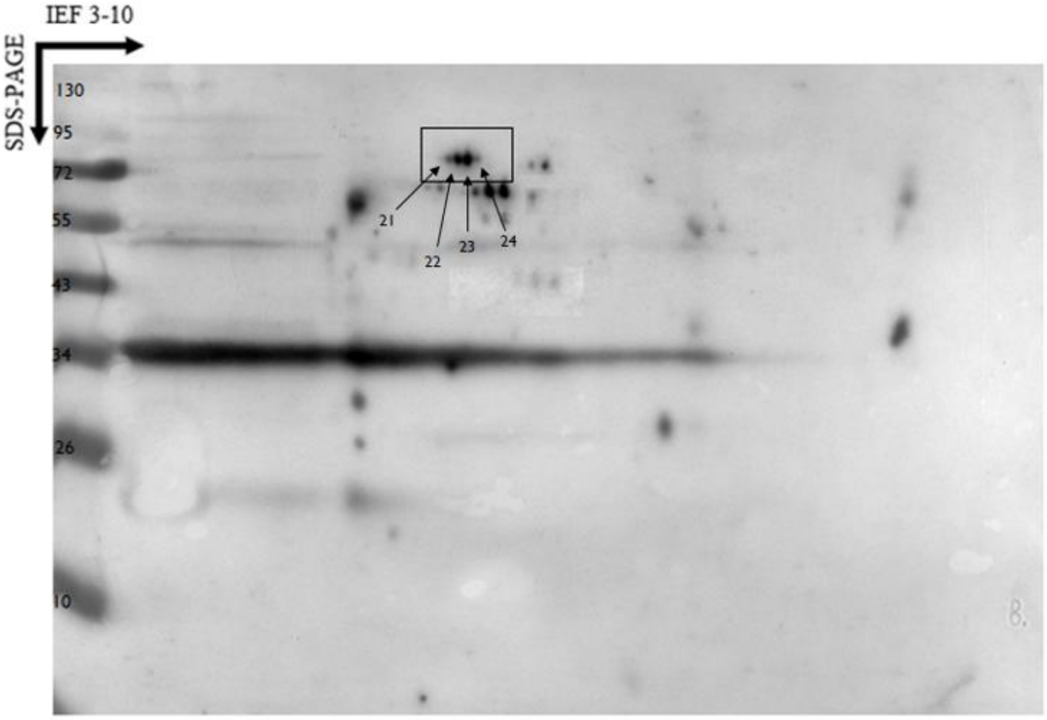

Patient 14BB
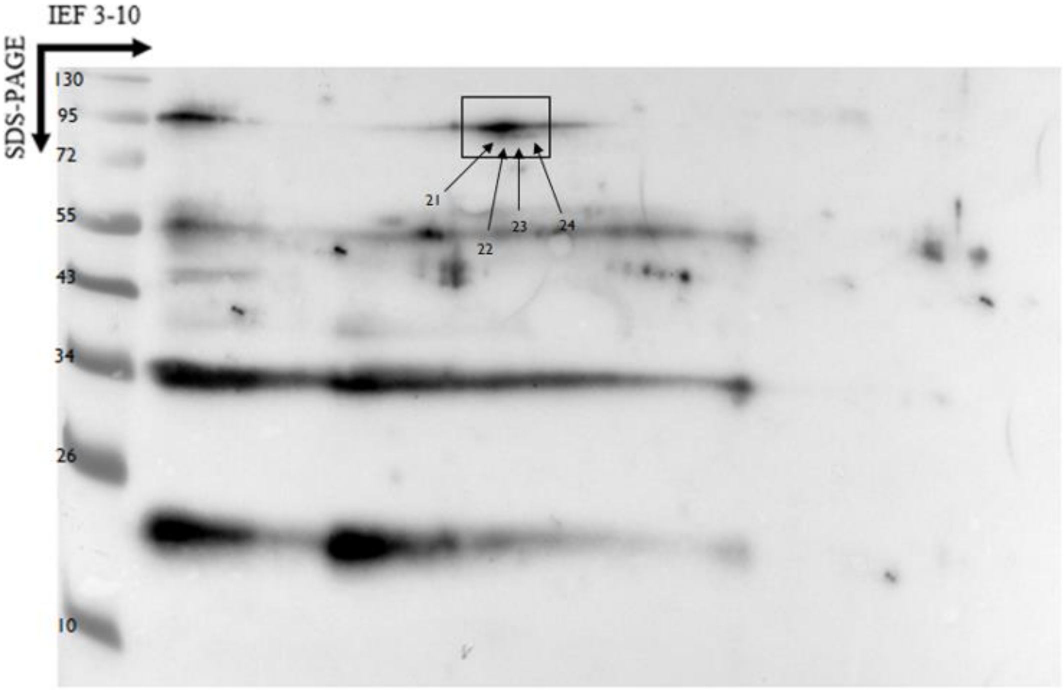

Patient 15BB
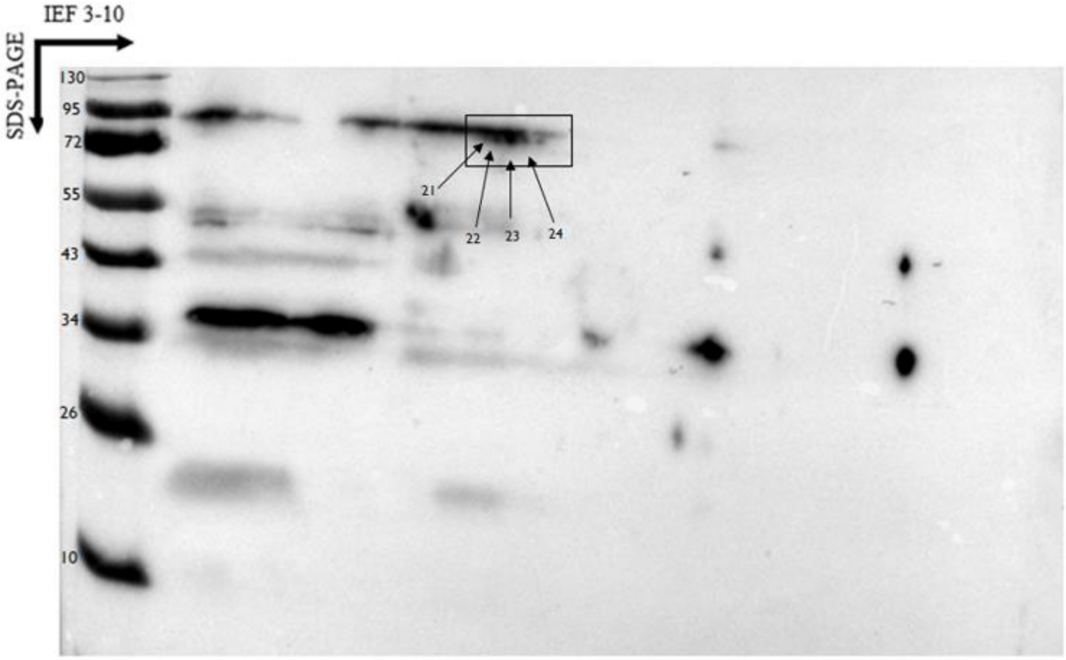

Patient 16BB
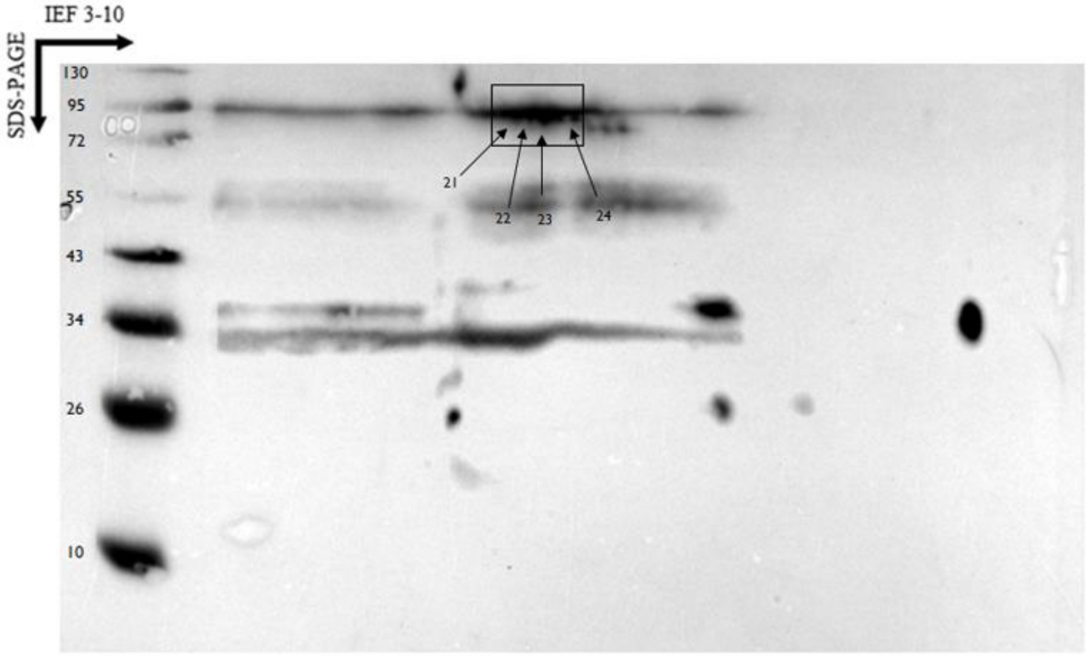

Patient 17BB

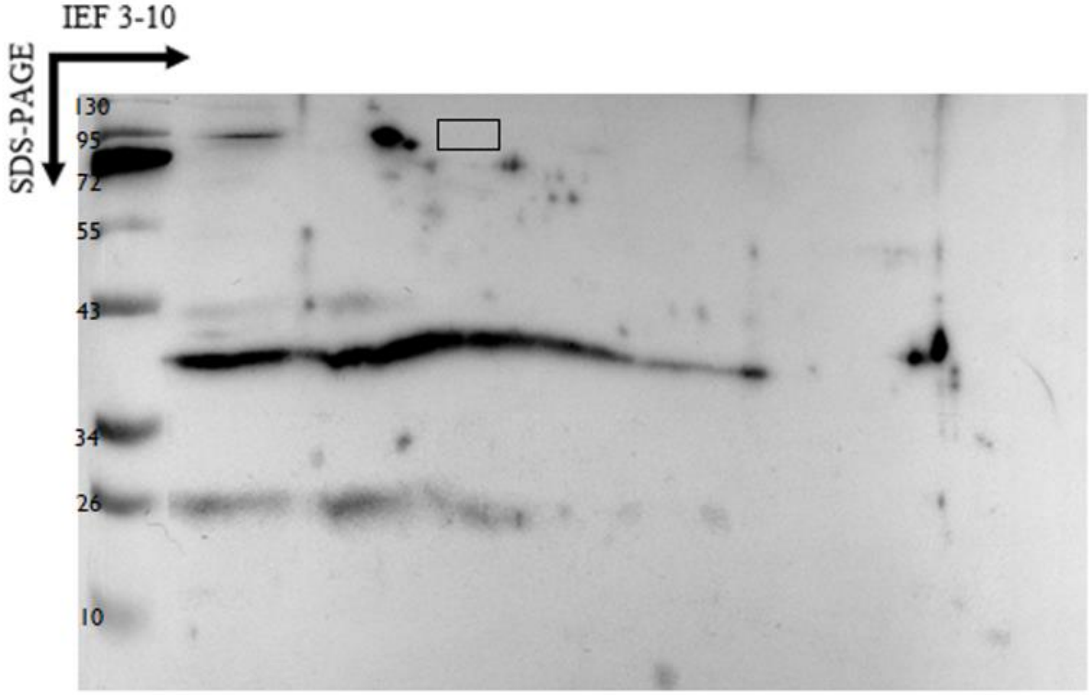

Patient 18BB

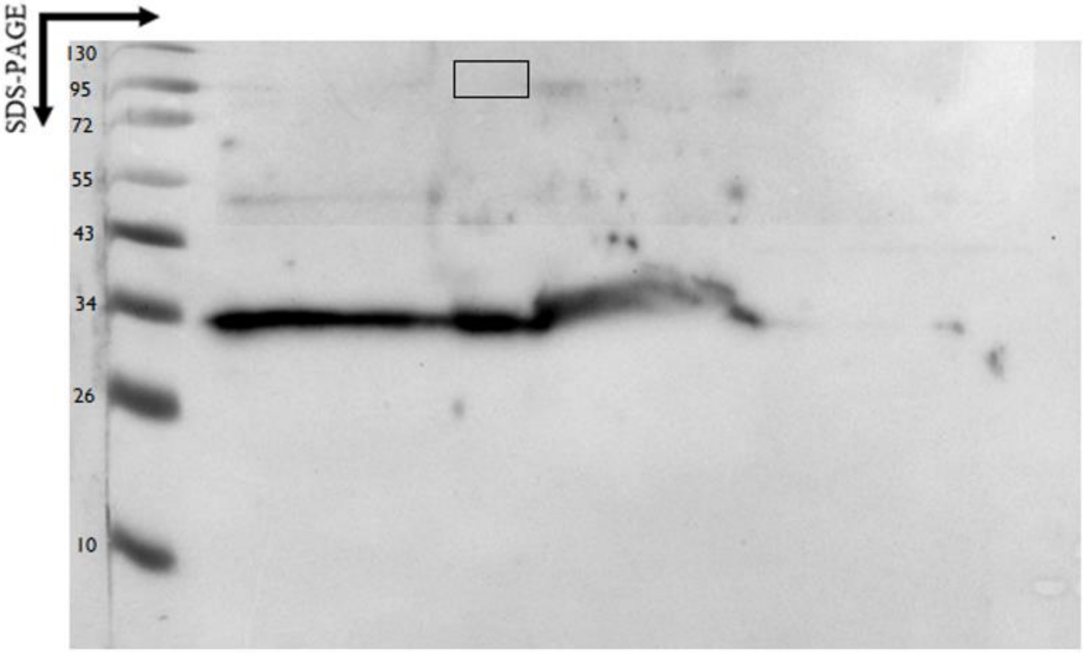

Patient 19BB

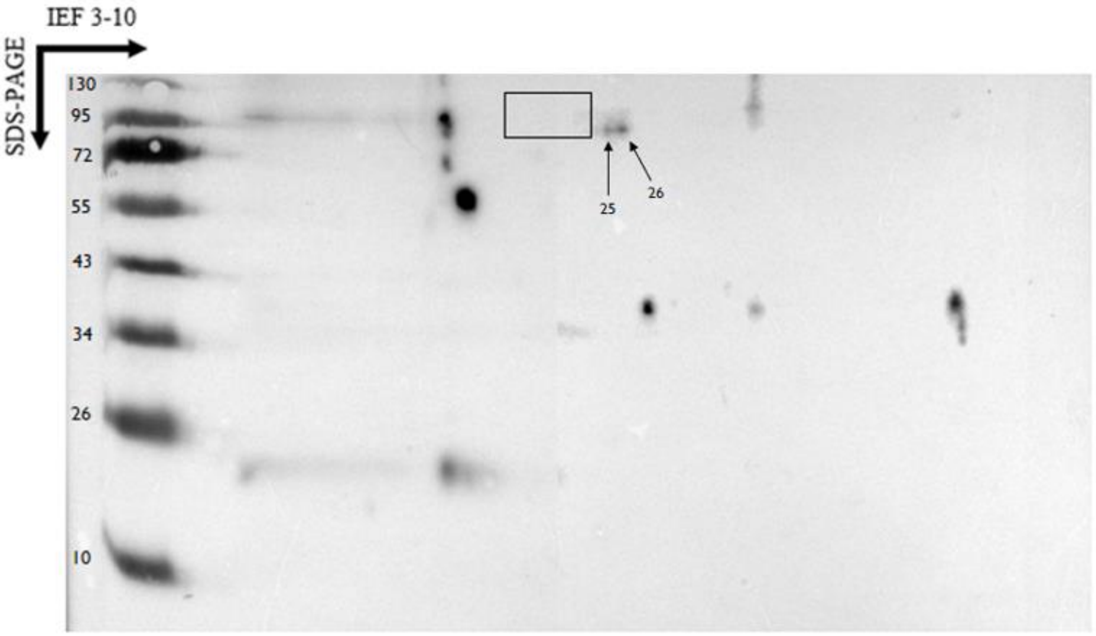

Supplementary data S2. Immunoblot obtained using sera positive for the IgM triplet (NNTPos ; Patients 1BB to 16BB) or negative for this same IgM triplet (NNTNeg ; Patients 17BB to 19BB). The black box indicates the location of spots 21 to 24 only found on NNTPos sera and marked by arrows when present. The immunoblot obtained with the NNTNeg sera from the patient 19BB shows spots 25 and 26 which were excluded because they were not specific to the IgM triplet.
